# Capitalizing on the heterogeneous effects of CFTR nonsense and frameshift variants to inform therapeutic strategy for cystic fibrosis

**DOI:** 10.1371/journal.pgen.1007723

**Published:** 2018-11-16

**Authors:** Neeraj Sharma, Taylor A. Evans, Matthew J. Pellicore, Emily Davis, Melis A. Aksit, Allison F. McCague, Anya T. Joynt, Zhongzhu Lu, Sangwoo T. Han, Arianna F. Anzmann, Anh-Thu N. Lam, Abigail Thaxton, Natalie West, Christian Merlo, Laura B. Gottschalk, Karen S. Raraigh, Patrick R. Sosnay, Calvin U. Cotton, Garry R. Cutting

**Affiliations:** 1 McKusick-Nathans Institute of Genetic Medicine, Johns Hopkins University School of Medicine, Baltimore, Maryland, United States of America; 2 Division of Pulmonary and Critical Care Medicine, Department of Medicine, Johns Hopkins Hospital, Baltimore, Maryland, United States of America; 3 Departments of Pediatrics, Physiology and Biophysics, Case Western Reserve University, Cleveland, Ohio, United States of America; Stanford University School of Medicine, UNITED STATES

## Abstract

CFTR modulators have revolutionized the treatment of individuals with cystic fibrosis (CF) by improving the function of existing protein. Unfortunately, almost half of the disease-causing variants in *CFTR* are predicted to introduce premature termination codons (PTC) thereby causing absence of full-length CFTR protein. We hypothesized that a subset of nonsense and frameshift variants in *CFTR* allow expression of truncated protein that might respond to FDA-approved CFTR modulators. To address this concept, we selected 26 PTC-generating variants from four regions of *CFTR* and determined their consequences on CFTR mRNA, protein and function using intron-containing minigenes expressed in 3 cell lines (HEK293, MDCK and CFBE41o-) and patient-derived conditionally reprogrammed primary nasal epithelial cells. The PTC-generating variants fell into five groups based on RNA and protein effects. Group A (reduced mRNA, immature (core glycosylated) protein, function <1% (n = 5)) and Group B (normal mRNA, immature protein, function <1% (n = 10)) variants were unresponsive to modulator treatment. However, Group C (normal mRNA, mature (fully glycosylated) protein, function >1% (n = 5)), Group D (reduced mRNA, mature protein, function >1% (n = 5)) and Group E (aberrant RNA splicing, mature protein, function > 1% (n = 1)) variants responded to modulators. Increasing mRNA level by inhibition of NMD led to a significant amplification of modulator effect upon a Group D variant while response of a Group A variant was unaltered. Our work shows that PTC-generating variants should not be generalized as genetic ‘nulls’ as some may allow generation of protein that can be targeted to achieve clinical benefit.

## Author summary

The development of variant specific modulators that correct dysfunctional cystic fibrosis transmembrane conductance regulator (CFTR) protein is an excellent example of precision medicine. Currently there is no molecular treatment available for individuals with cystic fibrosis (CF) carrying nonsense or frameshift variants because such variants introduce a premature termination codon (PTC), and are not expected to produce CFTR protein. We have performed a systematic study of nonsense and frameshift variants located in four regions of *CFTR* that we postulated should have varying effects on mRNA stability, protein production, and/or function. Using primary nasal cells and three different cell line models stably expressing CFTR expression mini-genes (EMGs), we report molecular consequences of 26 PTC-generating variants in *CFTR*, and identify which variants allow generation of CFTR protein that is responsive to currently available modulator therapies and which require alternative therapeutic approaches.

## Introduction

The development of variant-specific modulators that correct dysfunctional cystic fibrosis transmembrane conductance regulator (CFTR) protein is an excellent model for precision medicine [1–4]. Cystic fibrosis (CF) is a progressive, multi-organ, life-threating, autosomal recessive disease caused by variants in *CFTR* gene leading to reduced or no protein function in approximately 70,000 individuals worldwide [5–7]. Two classes of compounds have been approved by the US Food and Drug Administration (FDA). Ivacaftor (VX-770; Kalydeco) potentiates function by increasing the probability of channel opening to enhance chloride ion conductance of *CFTR* gating variants [8, 9]. Lumacaftor (VX-809) corrects the processing and trafficking of the most common CF-causing variant (F508del) to increase the quantity of CFTR channels at the cell surface [10]. A potentiator-corrector combination (ivacaftor and lumacaftor; Orkambi) has been approved for individuals with CF who carry two copies of the F508del variant [11]. More recently, a new CFTR corrector, tezacaftor (VX-661) in combination with ivacaftor (Symdeko) has demonstrated clinical efficacy in individuals who carry two copies of F508del, or one copy of F508del and a variant from a select set of ‘residual function variants’ [12–14]. While these break-through treatments dramatically alter outcomes in CF, they require the presence of targetable CFTR protein. However, approximately 28% of individuals with CF carry one or two variants that introduce premature termination codons (PTCs) resulting in loss of CFTR protein (https://cftr2.org).

The challenge of treating variants that cause premature termination is not unique to CF. It has been estimated that one-third of inherited and acquired human diseases are caused by nonsense, frameshift or splice-site variants that lead to generation of PTCs [15]. RNA transcripts bearing a PTC are generally targeted for elimination by a cellular quality-control mechanism called nonsense-mediated mRNA decay (NMD) [16]. Our current mechanistic understanding of NMD leads to the prediction that transcripts containing PTCs greater than 50 nucleotides upstream of the last exon-exon junction should undergo NMD [17]. In cells derived from individuals carrying PTC-generating variants, NMD reduces the level of mRNA transcripts to 5–25% of normal (i.e. PTC-free) level and substantially reduces synthesis of the encoded truncated protein [18]. Moreover, truncated proteins that derive from any residual nonsense transcripts typically lack function. However, there are circumstances where PTC-generating variants could produce transcript resulting in functional protein. Nonsense or frameshift variants within the last exon generally do not activate NMD thereby allowing synthesis of C-terminally truncated polypeptides [17, 19]. Furthermore, the efficiency of NMD can vary among cell types and individuals [20, 21]. Consequently, transcripts containing PTCs, even those that are targeted by NMD, can be maintained at low steady-state levels that may allow production of truncated protein [22–26]. Under both scenarios, protein may be present in cells that can be targeted with small molecules to generate sufficient levels of function to ameliorate disease. However, for many genes, including *CFTR*, it is unknown which, if any, PTC-generating variants permit production of protein that is targetable.

At least one intron, and pre-mRNA splicing are required for NMD of mammalian mRNAs that harbor or acquire PTCs [27, 28]. Inclusion of at least 200 bp intron sequences each from the 5' and 3' splice sites ensures that majority of the regulatory signals necessary for constitutive and alternative splicing are present [29, 30]. We and others have demonstrated that expression minigenes (EMGs) containing at least 200 bp of flanking intron splices heteronuclear pre-mRNA in precisely the same fashion, and with the same fidelity as observed in primary cells [31, 32]. Furthermore, we have shown that disease-associated variants alter splicing patterns of EMGs that replicate patterns found in primary nasal cells of affected individuals [33].

Here, we have performed a systematic study of nonsense and frameshift variants located in four regions of *CFTR* that were postulated to have varying effects on mRNA stability, protein production, and/or function. Using primary nasal cells and three different cell line models stably expressing *CFTR-*EMGs, we report molecular consequences of 26 PTC-generating variants in *CFTR*, and identify which variants allow generation of CFTR responsive to currently available modulator therapies and those that require alternative therapeutic approaches.

## Results

### Nonsense and frameshift variants in the 3’ region generate modulator responsive CFTR protein

**Fig 1:**
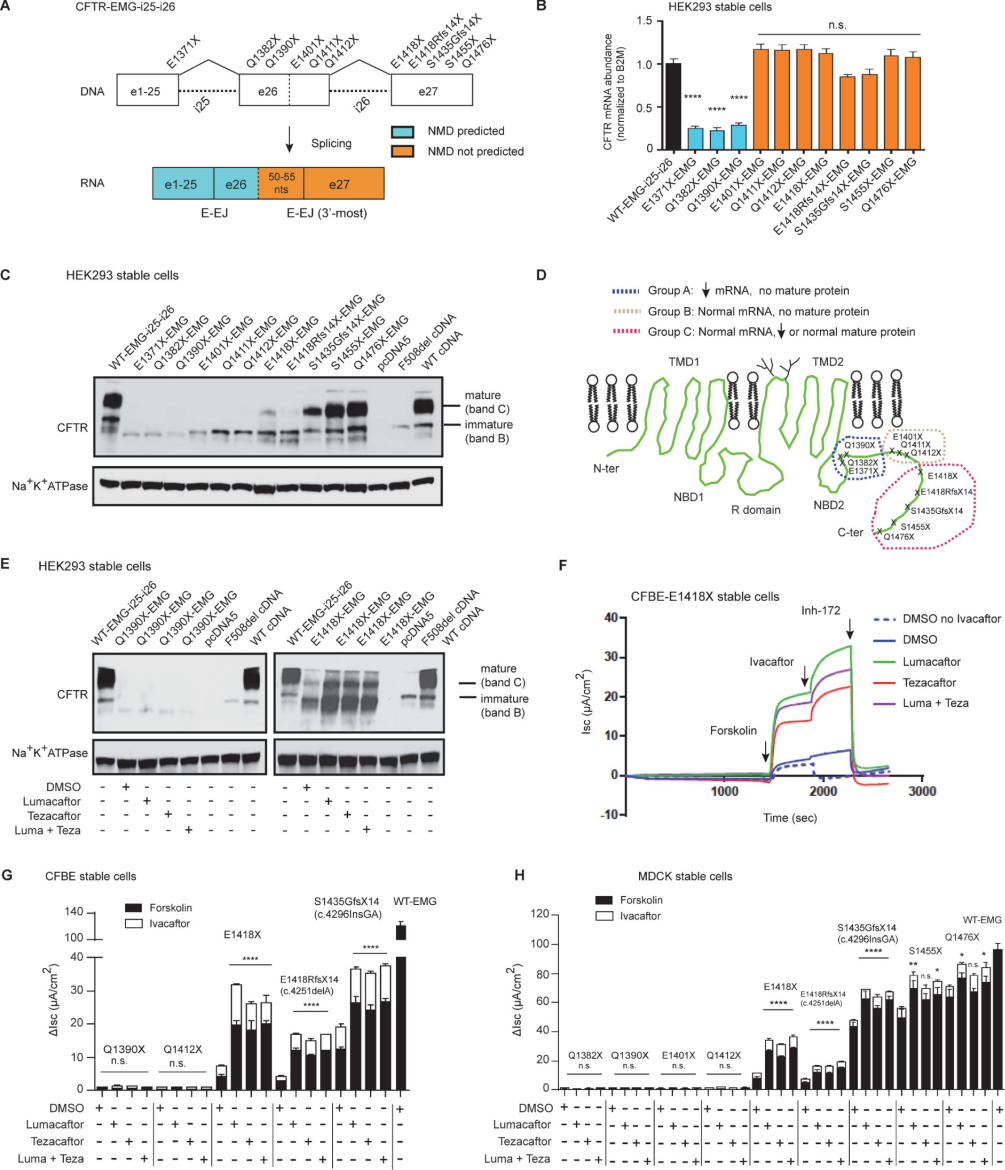
Nonsense and frameshift mutations at 3’ region that synthesize complex glycosylated truncated protein can respond to CFTR modulators. **(A)** Schematic of CFTR-Expression Minigene with full-length introns 25 and 26 (EMG-i25-i26) constructed in pcDNA5FRT plasmid. CFTR expression is driven by a CMV promoter. The location of each studied variant is shown relative to *CFTR* exons and regions predicted to elicit NMD. **(B)** Real-time quantitative reverse transcription polymerase chain reaction (RT-qPCR) showing relative steady state levels of *CFTR* transcript in HEK293 stable cells expressing wild-type EMG or EMGs with nonsense or frameshift variants, as indicated. Values were normalized to *B2M*. Mean ± SEM (*n* = 3) measured in triplicates. *P* value was determined by one way ANOVA. **** indicates significant difference (*P*≤0.0001) when compared with *CFTR* mRNA abundance in cells expressing WT-EMG. **(C)** Immunoblot (IB) of the steady state amounts of immature core-glycosylated (*band B*) and the mature complex-glycosylated mature CFTR protein (*band C*). Lysates were collected from HEK293 cells expressing WT-EMG or EMGs with different PTC-generating variants. Lysates from cells expressing either intronless WT CFTR or F508del served as controls, or empty vector as negative control. 40 μg of total cell lysates were electrophoresed and IB was probed with anti-CFTR antibody (596 # Cystic Fibrosis Foundation Therapeutics). **(D)** Schematic illustration showing three groups of 3’- nonsense variants based on mRNA stability and protein maturity. **(E)** Immunoblot of HEK293 stable cells expressing Q1390X or E1418X. The cells were incubated for 48 h with DMSO (.03%) or corrector compounds (lumacaftor and tezacaftor either alone or in combination—3 μM each). CFTR was visualized with anti-CFTR antibody, 596 (CFFT). **(F)** A representative Ussing chamber tracing of EMG E1418X-expressing CFBE stable cells grown on snap-wells. Short-circuit current (I_sc_) measurements were recorded in Ussing chambers after treatment of cells with 0.03% DMSO (vehicle) or 3 μM corrector compounds (lumacaftor/tezacaftor or both) for 48 h. Cells were mounted on Ussing chambers to measure CFTR mediated chloride channel activity as a proxy of CFTR function. After stabilization of the basal current, forskolin (10 μM) was added to the basolateral chamber followed by potentiator, ivacaftor (10 μM), and CFTR Inhibitor 172 (10 μM) added to the apical chambers. Inh-172 was added earlier in DMSO no ivacaftor (dashed blue line) treated cells. **(G and H)** Stacked bar graphs indicate effect of modulator treatment on CFBE **(G)** and MDCK **(H)** stable cells expressing different CFTR 3’ nonsense variants. Change in I_sc_ (ΔIsc) was defined as the current inhibited by Inh-172 after sustained Isc responses were achieved upon stimulation with forskolin alone or sequentially with ivacaftor. Mean ± SEM (*n* = 3–8). WT-CFTR function represents forskolin stimulated Isc without modulator treatment in cells expressing EMG i25-i26. *P* value was determined by one way ANOVA. **** *P* ≤0.0001, ** *P* ≤0.01, * *P*≤0.05, and n.s. (not significant, *P*>0.05); when compared with forskolin stimulated CFTR function in DMSO (vehicle) treated cells expressing respective variant.

Variants that introduce premature termination codons (PTCs) located in the last exon or less than 50–55 nucleotides upstream of the 3’-most exon-exon junction (E-EJ) generally do not elicit nonsense-mediated mRNA decay (NMD) [17]. Consequently, individuals carrying such variants have stable RNA transcripts that can synthesize C-terminal truncated protein [34]. As we know that certain 3’ nonsense variants in *CFTR* generate stable protein [35–37], we wanted to determine if the function of these truncated proteins could be augmented by CFTR modulators [13]. To test this concept, we used expression mini-genes (EMG) to evaluate the transcriptional and translational effects of eleven variants (nine nonsense and two frameshift variants) that introduce a PTC into the most distal 3’ region of *CFTR* (**Fig 1A,** top). EMGs contain full-length *CFTR* cDNA and introns flanking the variants under study, and they faithfully reproduce splicing patterns observed in affected tissues (**S1 Fig**) [32, 33]. A key advantage of EMGs is the inclusion of intron sequences which allow formation of E-EJs upon splicing that are required to engage NMD (**Fig 1A, bottom**)[27]. EMGs containing each of the 11 variants and wild-type (WT) were individually integrated into a single genomic site in Human Embryonic Kidney (HEK) 293-Flpin cells. Six nonsense variants (E1401X to Q1476X), and two frameshift variants (p.E1418RfsX14 and p.S1435Gfs14X) predicted to evade NMD based on their location (**Fig 1A**) demonstrated no significant difference in mRNA levels compared to WT CFTR **(Fig 1B)** [38]. Conversely, the three variants located in regions predicted to be subjected to NMD (E1371X, Q1382X and Q1390X) had significantly reduced levels of CFTR mRNA transcript consistent with degradation. The presence of detectable amounts of PTC-bearing transcript is expected as NMD is not completely effective in cell line models that utilize potent constitutive promoters (as employed here). Other investigators have reported that ~5–25% of PTC bearing mRNA can escape NMD under these circumstances [18, 39].

EMGs offer the advantage that protein synthesis can be studied simultaneously with RNA synthesis and splicing [32]. The WT-CFTR-EMG produced abundant mature, complex-glycosylated CFTR protein (band C) and minor amounts of immature, core-glycosylated CFTR protein (band B) as determined by immunoblot (IB) analysis **(Fig 1C)**. Variants at or between codons 1371 and 1412 generated only immature truncated protein (**Fig 1C, S2 Fig**). In contrast, variants located more 3’ including nonsense (E1418X), and two frameshifts (E1418Rfs14X and S1435Gfs14X) that truncate protein at residue 1442 and 1459 respectively generated minimal to moderate amounts of mature truncated protein. The final two variants S1455X and Q1476X, showed no apparent effect on the steady-state amounts of the immature or mature truncated forms of CFTR when compared with WT, as shown previously (**Fig 1C, S2 Fig)** [40, 41]. The nonsense and frameshift variants in the 3’region fell into three groups (A-C) based on effects on RNA and protein levels (**Fig 1D**). We used this molecular characterization to select variants for testing with FDA-approved CFTR corrector compounds. Variant Q1390X was chosen to determine if any protein synthesized from the severely reduced levels of RNA transcript could be stabilized. However, treatment of cells expressing the Q1390X-CFTR-EMG with correctors either alone or in combination (lumacaftor/tezacaftor or both) did not result in the appearance of CFTR protein (left panel, lanes 3–5 vs lane 2) (**Fig 1E)**. Conversely, the correctors (lumacaftor/tezacaftor or both) increased the abundance of mature and immature CFTR in the cell line expressing E1418X-EMG (right panel, lanes 3–5 vs lane 2; **Fig 1E)**.

To evaluate the function of C-terminal truncated forms of CFTR, EMGs were integrated into the genomes of CF bronchial epithelial cells (CFBE41o-) and/or Madin-Darby Canine Kidney (MDCK) cells. These two cell lines retain the ability to polarize and each has been used previously to assess CFTR chloride channel function [42–50]. Chloride channel activity of CFTR was measured in Ussing chambers by activation with forskolin followed by inhibition with the CFTR-specific compound Inh-172. A representative tracing from CFBE cells expressing E1418X-EMG (**Fig 1F,** dashed blue line) from group C demonstrates that CFTR chloride channel function is present (ΔI_sc_ = 4.1 ± 0.6 μA/cm^2^ representing ~ 3.5% of the chloride channel current generated in cell lines expressing WT-CFTR, **Fig 1G**). Acute exposure to the potentiator (ivacaftor) resulted in a minimal increase in function (**Fig 1F**, blue solid line). However, CFTR correctors (lumacaftor, tezacaftor or both) in combination dramatically increased E1418X-CFTR current by ~ 4.6 fold equivalent to ~ 16% of WT-CFTR function, consistent with the increased steady state levels of mature protein upon treatment with these correctors (**Fig 1E**; green, red, and purple lines). Addition of ivacaftor to the corrector-treated cells further increased current achieving about 23% of the function of cells expressing WT-CFTR (**Fig 1F and 1G**). Likewise, two frameshift variants in group C (E1418RfsX14 and E1435GfsX14) exhibited improvement in CFTR function upon modulator treatments (~ 2.3% to ~ 13.4% and ~ 10.4% to ~ 30.3% of WT-CFTR respectively) (**Fig 1G**). A variant in group B (Q1412X) generated minimal CFTR function, and exhibited no improvement with modulators (**Fig 1G**). Similarly, Q1390X from group A displayed negligible function and no response to modulators, as expected for a variant that induced NMD leading to severe reduction of RNA and no mature CFTR protein (**Fig 1G**). To address whether cell-type specific factors affected processing, function or response of truncated forms of CFTR, we created MDCK cells expressing 9 of the 11 variants. Group C variants E1418X and E1418Rfs14X exhibited residual channel activity (Isc = 8.8 ± 0.6 μA/cm^2^ and 5.5 ± 0.3 μA/cm^2^ representing ~ 9% and ~ 6% of WT-CFTR function respectively) (**Fig 1H**). This level of function is consistent with that reported from studies of primary nasal cells bearing E1418X variant (~ 10% of WT) [51] and is also consistent with minimal amount of mature CFTR protein generated by this variant (**Fig 1C**). Lumacaftor, tezacaftor or both followed by acute treatment with ivacaftor increased CFTR current in the MDCK cell lines expressing E1418X and E1418Rfs14X to ~ 35% and ~ 18% of WT-CFTR function, respectively. Likewise, modulator treatments increased CFTR function to near WT levels in the cells expressing three downstream variants from group C (E1435Gfs14X, S1455X and Q1476X) (**Fig 1H**). As expected, three upstream nonsense variants in group A (Q1382X, Q1390X) and group B (E1401X and Q1412X) displayed negligible function (<1% of WT-CFTR), and no improvement with modulators. These results indicate that Group C nonsense and frameshift variants downstream of codon 1417 allow synthesis of stable truncated CFTR that responds to CFTR modulators.

### Nonsense variants in exon 22 generate modulator responsive CFTR protein

**Fig 2:**
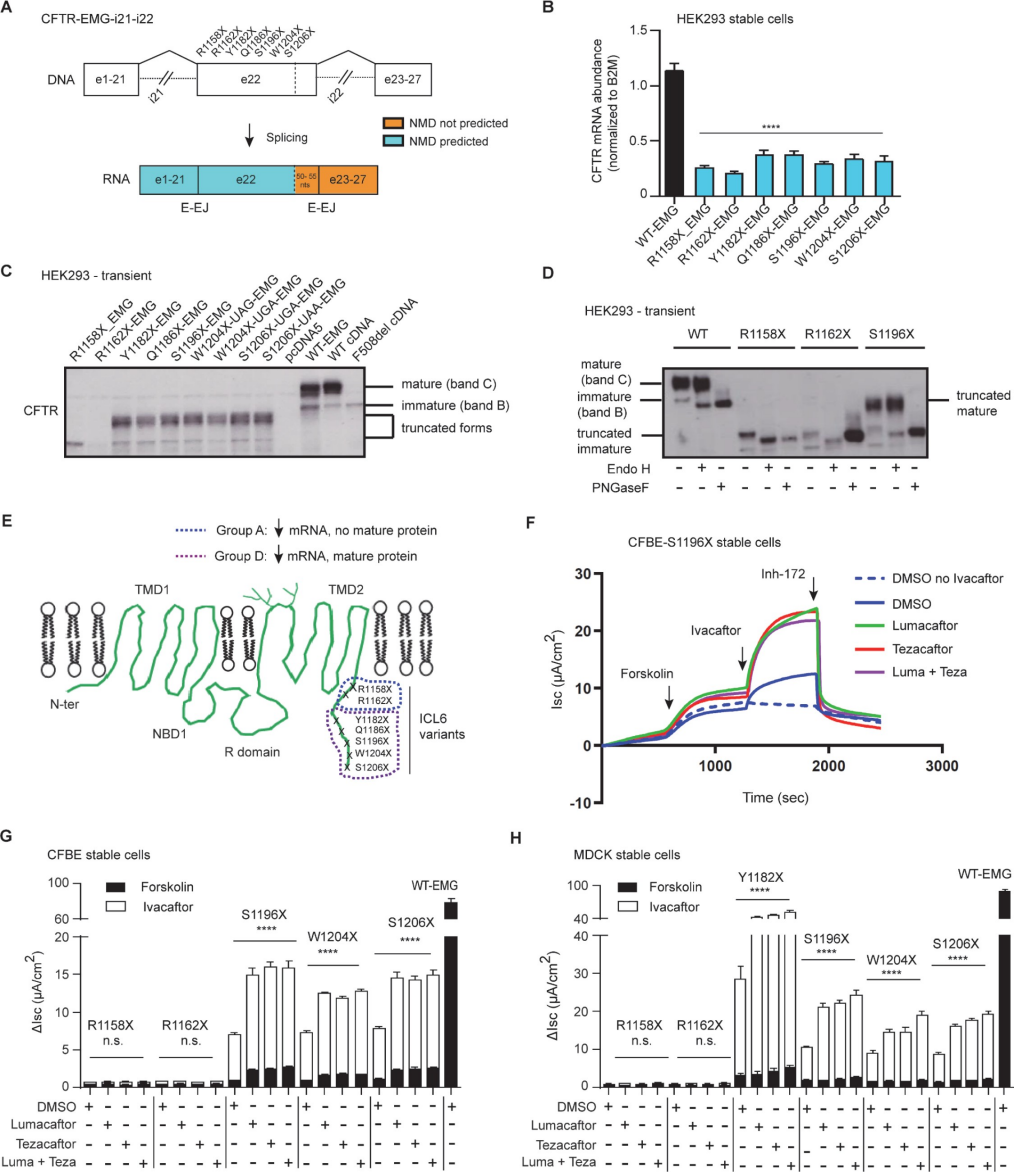
Corrector treatment increases CFTR activation response of nonsense variants in exon 22 that result in mature truncated CFTR. **(A)** Schematic of CFTR-Expression Minigene with abridged introns 21 and abridged intron 22 (EMG-i21-i22) constructed in pcDNA5FRT plasmid. CFTR exons are shown in boxes and two abridged introns in dashed lines. The location of each studied variant is shown relative to the *CFTR* exons and regions predicted to elicit NMD. **(B)** RT-qPCR showing relative steady state levels of *CFTR* transcript in HEK293 stable cells expressing wild-type EMG or EMGs with truncations at residue position, as indicated on the labels. Values were normalized to *B2M*. Mean ± SEM (*n* = 3) measured in triplicates. *P* value was determined by one way ANOVA. **** indicates significant difference (*P*≤0.0001) when compared with *CFTR* mRNA abundance in cells expressing WT-EMG. **(C)** Steady state levels of CFTR protein from HEK293 cells transiently transfected with wild-type EMG or EMGs with different nonsense variants. 40 μg of total cell lysates were electrophoresed and IB was probed with anti-CFTR antibody-MM13-4 (EMD Millipore). **(D)** Representative IB showing sensitivity of CFTR to PNGaseF and Endo H. Mature complex glycosylated band is sensitive to PNGase only, whereas immature core glycosylated band is sensitive to both PNGase and EndoH. **(E).** Schematic illustration of the nonsense variants in the protein context showing their classification into two groups based on mRNA stability and protein maturity. Each nonsense variant truncates CFTR at intracellular loop 6 (ICL6) just before NBD2. **(F)** A representative Ussing chamber tracing of CFBE cells stably expressing S1196X-EMG. Short-circuit (I_sc_) measurements were recorded in Ussing chambers after treatment of cells with 0.03% DMSO (vehicle) or 3 μM corrector compounds (lumacaftor/tezacaftor or both) for 48 h. (**G and H)** Stacked bar graphs indicate effect of modulator treatment on CFBE **(G)** and MDCK **(H)** stable cells expressing different CFTR 3’ nonsense variants. Change in I_sc_ (ΔIsc) was defined as the current inhibited by Inh-172 after sustained Isc responses were achieved upon stimulation with forskolin alone or sequentially with ivacaftor. Mean ± SEM (*n* = 3–8). WT-CFTR function represents forskolin stimulated I_sc_ without modulator treatment in cells expressing EMG i21-i22. *P* value was determined by one way ANOVA. **** indicates significant difference (*P* ≤0.0001), n.s. (not significant, *P*>0.05); when compared with forskolin stimulated CFTR function in DMSO (vehicle) treated cells expressing respective variant.

Prior studies of synthetically truncated forms of CFTR revealed that protein sequence following intracellular loop (ICL6) is not required for conformational maturation of CFTR [52, 53]. To test whether naturally occurring CF-causing nonsense variants in exon 22 encoding ICL6 (codons 1150 to 1218) allow production of stable and potentially drug-targetable forms of truncated CFTR, we utilized EMG-i21-i22 that contains abridged introns 21 and 22 incorporated into full-length CFTR cDNA to evaluate variant effects (**Fig 2A**, top, **S3 Fig**). Each of the seven nonsense variants are predicted to engage NMD because they are located >50 nt from the 3’-most E-EJ within EMG-i21-i22 (**Fig 2A**, bottom). Indeed, each of the seven nonsense variants produced lower steady state levels of CFTR mRNA compared to WT (**Fig 2B**). Transient expression in HEK293 cells was utilized to determine if any translated products were processed to mature forms of CFTR; conditions that could lead to therapeutic benefit if NMD could be counteracted. IB revealed two apparent patterns: core glycosylated truncated CFTR only (R1158X and R1162X) or complex glycosylated and core glycosylated truncated forms of CFTR (7 variants; **Fig 2C**). To evaluate glycosylation status, proteins were subjected to endoglycosidase H which removes sugar moieties from immature core glycosylated protein (see EMG-WT, lane 2; **Fig 2D**) and PNGase F that removes sugars from mature complex and immature core glycosylated protein (EMG-WT, lane 3; **Fig 2D**). As expected, endoglycosidase endo H and PNGase F digestion altered truncated protein generated by EMGs bearing R1158X or R1162X (**Fig 2D**). Thus, only core glycosylated truncated protein was generated by these two variants. Conversely, susceptibility to digestion by PNGase F but not by endo H confirmed that the higher molecular mass CFTR protein generated by S1196X was complex glycosylated (**Fig 2D).** Complex glycosylated truncated CFTR generated by the remaining nonsense variants in this cluster showed the same susceptibility to PNGase F but not Endo H (**S4 Fig**). These results indicate that the nine naturally occurring nonsense variants in exon 22 fall into the previously described group A or group D based on mRNA abundance, and whether they permit synthesis of mature truncated CFTR (**Fig 2E**).

We next tested whether the mature truncated CFTR protein generated by group D nonsense variants were functional. CFBE cells stably expressing EMG-S1196X generated baseline chloride channel activity upon application of forskolin that was inhibited by inh-172 (**Fig 2F;** dashed blue line; I_sc_ = 1.03 ± 0.1 μA/cm^2^ representing ~1.3% of the chloride current generated by WT-CFTR in the same cell line). Acute exposure to ivacaftor generated a 4.5 fold increase in CFTR function from baseline levels (**Fig 2F**, blue solid line). There was only a marginal increase in forskolin stimulated CFTR function upon treatment of cells with correctors (lumacaftor, tezacaftor or both; **Fig 2F**; green, red, and purple lines). However, application of ivacaftor substantially increased CFTR function (~11 fold compared to baseline levels; **Fig 2F and 2G**). The level of combined modulator response of EMG-S1196X exceeded 10% of the chloride currents generated by cells expressing WT-CFTR. A similar profile of response was observed for cells expressing additional group D variants (W1204X and S1206X); whereas group A variants R1158X and R1162X failed to generate current in response to forskolin or after treatment with any of the CFTR modulators (**Fig 2G**), as observed for the Group A variants in the 3’ region.

To verify studies in CFBE cell lines, MDCK cell lines were created that stably expressed CFTR-EMGs bearing 6 of the exon 22 nonsense variants. EMG-S1196X generated functional CFTR protein with activity similar to that observed in CFBE cells (1.8 ± 0.3 μA/cm^2^ representing 1.7% of current observed in MDCK cells expressing WT-CFTR; **Fig 2G and 2H**). Furthermore, ivacaftor increased S1196X-CFTR function (~ 6.8 fold representing ~ 7.8% WT-CFTR); and application of correctors (lumacaftor, tezacaftor or both) resulted in dramatic increases in function (~ 12.5 fold representing ~ 21.5% WT-CFTR function; **Fig 2H**). Interestingly, group D variants W1204X and S1206X also exhibited similar robust responses when ivacaftor was combined with correctors (lumacaftor, tezacaftor or both; **Fig 2H**). Notably, Y1182X variant showed even greater response to the modulators when compared to other variants in group D (**Fig 2H**). Finally, as noted in CFBE cell lines, MDCK cells bearing EMGs with group A variants (R1158X and R1162X) did not generate forskolin-activated CFTR current or respond to any of the CFTR modulators (**Fig 2H**). Together, these results indicate that corrector and potentiator treatment, especially in combination, elicits substantial CFTR function for exon 22 nonsense variants that generate mature truncated CFTR.

### NMD inhibition has a synergistic effect on modulator treatment of exon 22 nonsense variants

**Fig 3:**
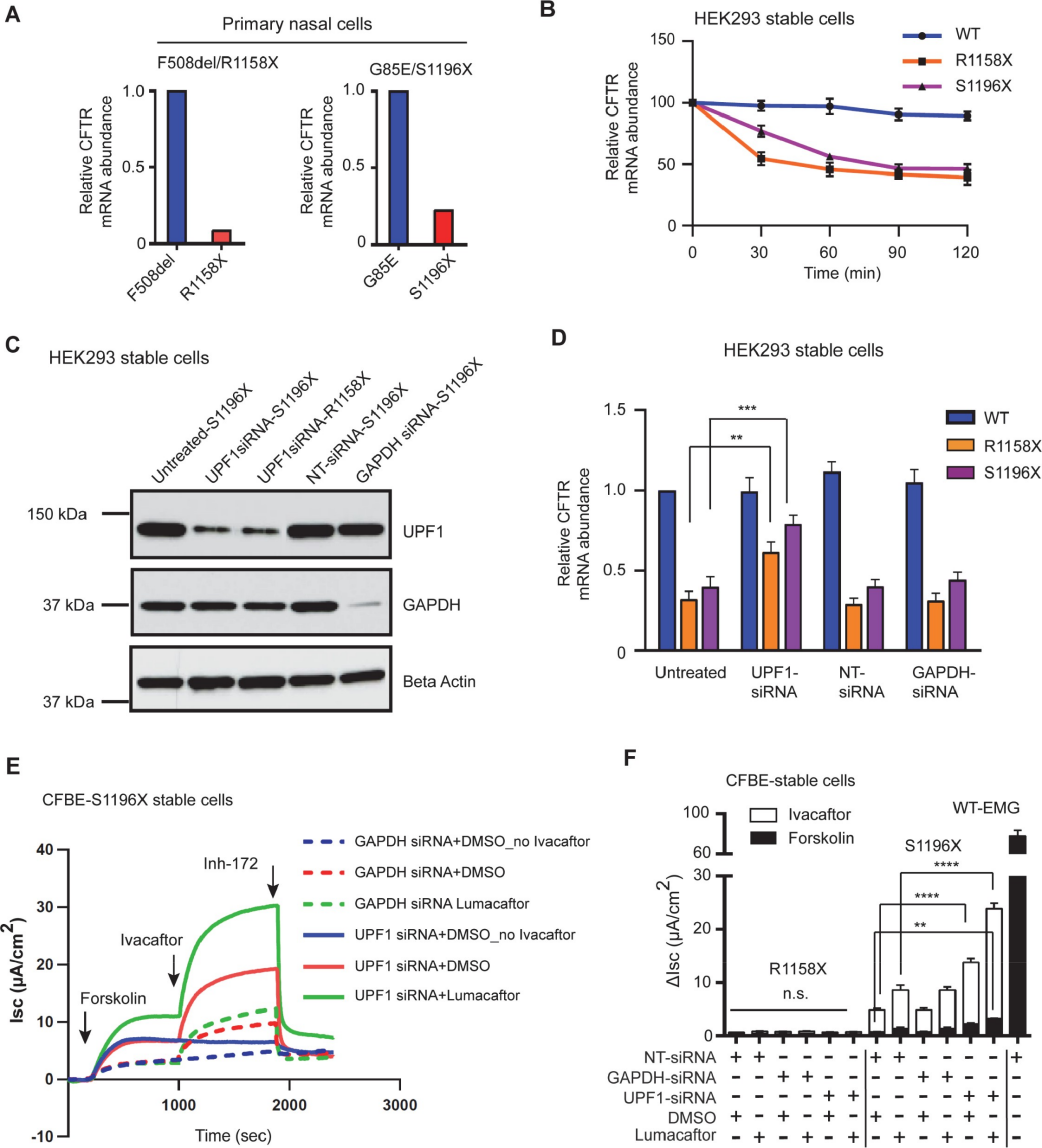
NMD inhibition has a synergistic effect on corrector-potentiator combination response in stable cells expressing nonsense variants in exon 22 that produce mature truncated CFTR. **(A)** Relative expression of the alternate *CFTR* allele in the primary nasal cells of CF individuals carrying exon 22 nonsense variant. Pyrosequencing assay was designed such that exon 22 with upstream and downstream flanking exons was amplified from the corresponding cDNA preparations. Sequencing primer yielded relative abundances of alternate alleles at the respective loci where nucleotide change occurred. **(B**) *CFTR* mRNA decay in HEK293 cells stably expressing wild type EMG or EMG harboring nonsense variants R1158X or S1196X. Actinomycin D (3 μg/ml) was added at time 0 to induce transcriptional shut-down. Cells were collected at the indicated time points. Levels of the *CFTR* mRNAs were assessed by RT-qPCR, normalized to B2M mRNA and displayed as a percentage of the levels at t = 0. Mean ± SEM (*n* = 3) **(C)** Efficiency of siRNA mediated knock down of *UPF1* detected on IB of whole cell lysates collected from HEK293 cells stably expressing either R1158X or S1196X. *GAPDH* siRNA and non-target (NT) siRNA were used as positive and negative controls respectively. Beta-Actin was used as loading control. **(D)** Effect of direct NMD inhibition on the level of *CFTR* transcript by siRNA mediated knock down of *UPF1* in HEK293 cells stably expressing either R1158X or S1196X. Levels of the *CFTR* mRNAs were assessed by RT-qPCR and normalized to *B2M* mRNA. Mean ± SEM, *n* = 3 independent biological triplicates, *P* value was determined by two way ANOVA. ** (*P*≤0.01) and *** (*P*≤0.001) indicate significant difference when compared with CFTR mRNA abundance in untreated cells **(E)** Short-circuit (I_sc_) tracings of CFBE-S1196X stable cells recorded in Ussing chambers after direct inhibition of NMD by *UPF1*. Cells were transfected with *Upf1* siRNA at 50% confluency for 4 days before being mounted on Ussing chambers. *GAPDH* and non-targeted (NT) siRNA transfections were used as controls. Cells were incubated with lumacaftor (3 μM) or DMSO (0.03%) during last 48h of siRNA transfections. **(F)** Stacked bar graphs indicate effect of UPF1 siRNA in combination with CFTR modulators. Change in I_sc_ (ΔI_sc_) was defined as the current inhibited by Inh-172 after sustained Isc responses were achieved upon stimulation with forskolin alone or sequentially with ivacaftor. Mean ± SEM (*n* = 3). *P* value was determined by one way ANOVA. ** (*P*≤0.01) indicate significant difference when compared with forskolin stimulated CFTR function in NT siRNA transfected cells and **** (*P*≤0.0001) indicate significant difference when compared with ivacaftor activated CFTR function in NT siRNA transfected cells incubated with or without lumacaftor. WT-CFTR function represents forskolin stimulated ΔI_sc_ without modulator treatment in cells expressing EMG i21-i22.

Antagonism of NMD caused by nonsense variants that produce modulator responsive CFTR could provide substantial therapeutic benefit. To address this issue, we evaluated whether NMD inhibition increases the function of exon 22 nonsense variants expressed in primary nasal epithelial cells. As predicted by EMGs, *CFTR* transcript bearing two exon 22 nonsense variants (R1158X and S1196X) was reduced in the primary cells. Quantification by pyrosequencing revealed that *CFTR* transcript bearing R1158X was much less abundant (8.5%) compared to *CFTR* transcript with the F508del variant (**Fig 3A,** left bar graph). F508del transcript is expressed at approximately 83% of WT- CFTR transcript [31, 54], suggesting that R1158X levels were ~ 7.0% of WT level. Likewise, the level of *CFTR* transcript with S1196X was significantly lower (22.2%) compared to transcript bearing G85E (**Fig 3A**, right bar graph). Expression levels of S1196X relative to WT could not be drawn since expression of G85E relative to WT has not been established so far. To evaluate the time scale of NMD inhibition, a mRNA stability assay was performed on the HEK293 cell lines stably expressing R1158X and S1196X. While WT CFTR transcript level was stable over 120 minutes, transcripts bearing either nonsense variant were degraded to ~50% of WT levels by 30 min (R1158X) or 90 min (S1196X) (**Fig 3B**). To verify that the reduction in transcript abundance was due to NMD, we used siRNA mediated knockdown of UPF1, a gene that mediates nonsense transcript degradation [55]. Western blot analysis showed efficient siRNA mediated downregulation of UPF1 expression in HEK293 cells stably expressing R1158X (~29.4%), and S1196X (~29.8%) (**Fig 3C**). Transfection of S1196X expressing cells with Non-Targeted (NT) and *GAPDH*-targeted siRNA had no effect on UPF1 level (**Fig 3C**). UPF1 knockdown resulted in significant increases in CFTR transcript abundance in HEK293 cells stably expressing R1158X (1.9±0.5 fold) and S1196X (2.1±0.7 fold) compared to untreated cells or cells transfected with non-target (NT) or GAPDH siRNA (**Fig 3D**). We next determined whether inhibition of NMD augments modulator treatment of CFTR bearing exon 22 nonsense variants. Cells transfected with UPF1 siRNA exhibited significant potentiation of S1196X-CFTR function by ivacaftor (red solid line, 13.81 ± 0.6 μA/cm^2^ representing ~ 18% WT-CFTR function **Fig 3E and 3F**); that was further increased upon corrector (lumacaftor) treatment (green solid line, 23.9 ± 1.1 μA/cm^2^ representing 30% WT-CFTR function **Fig 3E and 3F**). However, UPF1 inhibition could not increase the function of R1158X-CFTR consistent with prior evidence that this form of CFTR is severely misfolded and non-responsive to modulators, even after improvement of transcript abundance by NMD inhibition (**Fig 3F**). Finally, transfection with *GAPDH* siRNAs did not alter the effect of ivacaftor alone or ivacaftor/lumacaftor combination compared to non-targeted (NT) siRNA transfected cells (**Fig 3E and 3F**). Collectively, these results indicate that suppression of NMD should be able to amplify modulator response of CFTR bearing exon 22 nonsense variants that generate complex glycosylated truncated CFTR.

### A nonsense variant in exon 15 that causes a splicing defect generates modulator responsive CFTR protein

**Fig 4:**
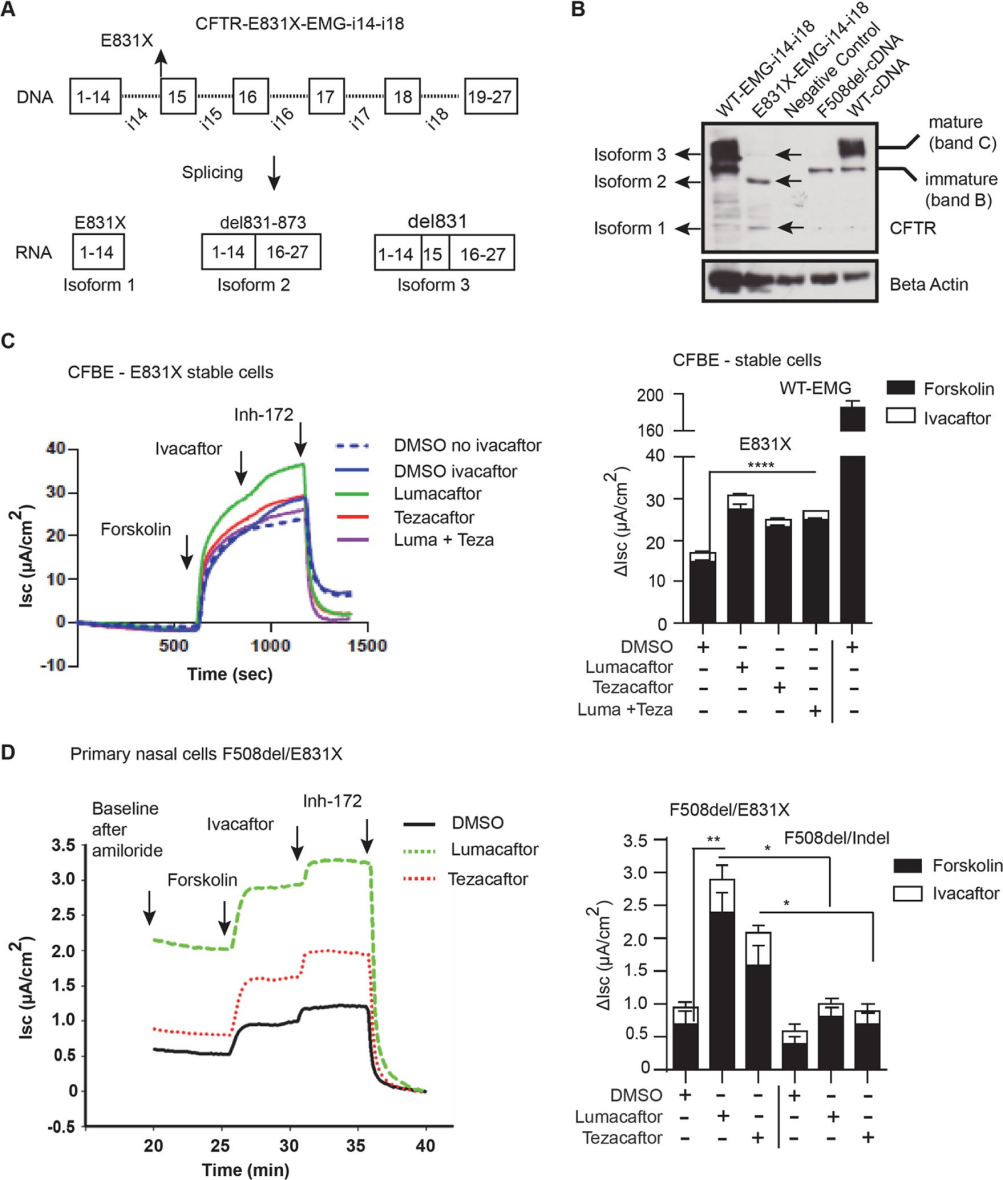
CFTR nonsense variant with splicing defect has residual function that benefits from modulator treatments. **(A)** A schematic illustration of CFTR-Expression Minigene with introns 14–18 (introns 14 and 16 are full-length, and 15, 17, & 18 are abridged). Arrow indicates location of E831X (top). CFTR mRNA splicing patterns of the total RNA extracted from HEK293 cells transiently transfected with E831X-EMG. **(B)** Steady state amounts of different isoforms of CFTR produced from E831X-EMG-i14-i18 expressed transiently in HEK293 cells. Lysates from cells expressing WT-EMG i14-i18, intronless WT CFTR or F508del served as positive controls, and empty vector as negative control. Immunoblot (IB) was probed with anti-CFTR antibody, 596 (CFFT). Horizontal arrows indicate to isoforms corresponding to (i) a normal codon (E831) substituted with a stop codon, (ii) deletion of complete exon 16, and (iii) deletion of a single amino acid E831. Beta-Actin was used as loading control. **(C)** Short-circuit (I_sc_) tracing of CFTR function observed in CFBE-stable cells expressing E831X mounted on Ussing chamber. Cells were treated for 48 h with correctors (lumacaftor/tezacaftor or both, 3 μM each) and acutely with potentiator (ivacaftor, 10 μM). Change in I_sc_ (ΔI_sc_) was defined as the current inhibited by Inh-172 after sustained Isc responses achieved upon stimulation with forskolin alone or sequentially with ivacaftor. Data are presented as mean±SEM (n = 3). *P* value was determined by one way ANOVA. **** indicates significant difference (*P* ≤0.0001) when compared with forskolin stimulated CFTR function in DMSO (vehicle) treated cells. WT-CFTR function represents forskolin stimulated Isc without modulator treatment in cells expressing EMG i14-i18. **(D**) A tracing of CFTR function observed in primary nasal epithelial cells of an individual harboring E831X/F508del. CF-Human nasal epithelial (HNE) cells were treated for 24 h with lumacaftor and tezacaftor, 3 μM each, and acutely with Ivacaftor (10 μM). Stacked bar graph is a comparison of improvement in CFTR function of E831X/F508del *vs* F508del/Indel. Alternate Indel alleles were either 2184insA, 2183delAA>G, or 3659del C. *P* value was determined by one way ANOVA. ** indicates significant difference (*P* ≤0.01) when compared with forskolin stimulated CFTR function in DMSO (vehicle) treated E831X/F508del HNEs. * (*P* ≤0.05) when compared with CFTR function in modulator treated F508del/Indel HNEs.

Nonsense variant E831X is caused by a change in the first nucleotide of exon 15 of *CFTR*. This change alters the 3’ splice site of intron 14 leading to the generation of aberrantly spliced *CFTR* transcripts in primary airway epithelial cells [56]. Of the resulting three proteins, only CFTR missing glutamate at codon 831 (CFTR-del831) generates mature glycosylated protein that functions similarly to WT [56]. However, response to CFTR modulators in cell-based system has not been reported for this ‘nonsense’ variant. To this end, we introduced E831X into an EMG containing flanking sequences from introns 14 to 18 (EMG-i14-18) (**Fig 4A**, top). WT-EMG-i14-i18 was found to splice normally in HEK293 stable cells (**S5 Fig**), as previously shown [32]. HEK293 stable cells expressing E831X-EMG generated 3 CFTR splice isoforms: Isoform 1 (CFTR-E831X i.e. truncation at 831), Isoform 2 (CFTR-del831-873 i.e. in frame deletion of exon 15), and Isoform 3 (CFTRdelE831, single amino acid deletion), (**Fig 4A**, bottom, and **S6 Fig**), as previously reported [56]. Furthermore, IB analysis identified three CFTR specific protein bands, each generated from their respective splice isoform (**Fig 4B**). Thus, the effect of E831X on mRNA splicing and protein production constitutes fifth group, E. To assess modulator response, CFBE cells stably expressing E831X-EMG were created to measure CFTR function. CFTR function was evident upon addition of forskolin and inhibition using inh-172 (14.7 ± 0.65 μA/cm^2^ representing ~8% of current in CFBE cells expressing WT-EMG) (**Fig 4C**, graph). Acute treatment with ivacaftor alone did not result in significant improvement of E831X-EMG function. However, correctors (lumacaftor, tezacaftor or both) increased E831X-CFTR function (~13.6% WT-CFTR) and subsequent acute addition of ivacaftor increased function further (~15.0% of WT-CFTR) (**Fig 4C**, graph). Furthermore, primary nasal epithelial cells harvested from an individual with CF harboring E831X (in *trans* with F508del) exhibited residual CFTR function (**Fig 4D**, black tracing) that was increased by ivacaftor and further augmented by correctors (lumacaftor and tezacaftor) (**Fig 4D**, green and red tracings). CFTR specific current that drops below baseline after addition of Inh-172 is due to the constitutive activation of CFTR in primary nasal cell culture. Of note, modulator responses were greater in F508del/E831X primary cells compared to F508del/Indel (2184InsA, 2183delAA>G, and 3659delC) primary cells (**Fig 4D**, graph). Since the indel variant does not generate functional CFTR, the increased CFTR function in the E831X/F508del cells compared to the indel/F508del cells can be attributed to CFTR generated by the E831X variant. Thus, modulator combinations demonstrate consistent evidence of functional improvement in primary and CFBE cells expressing E831X-CFTR.

### Nonsense variants in the 5’ region that naturally escape NMD can generate readthrough and modulator responsive CFTR protein

**Fig 5:**
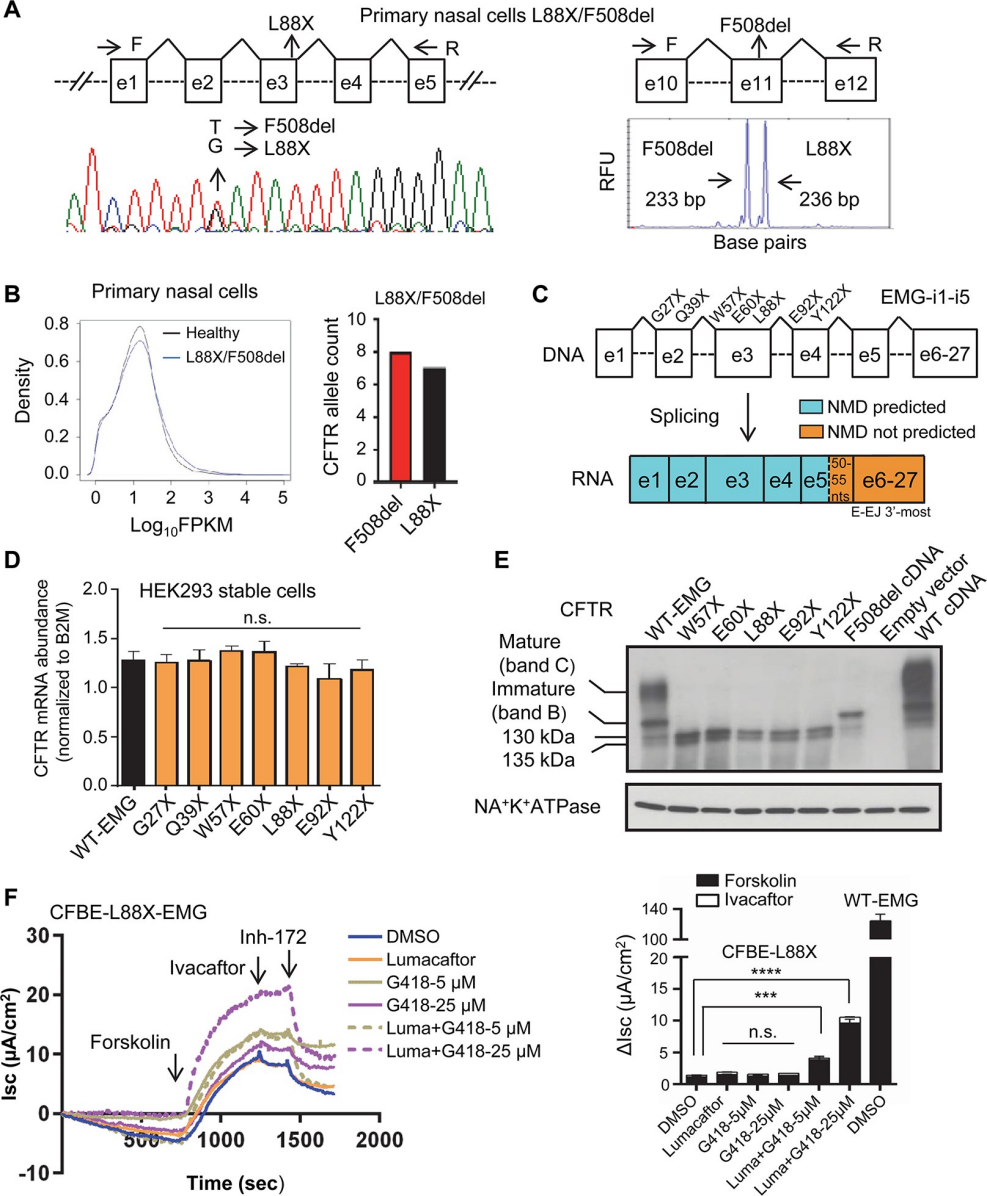
5’ nonsense variants that do not undergo NMD are the potential targets of read-through agents. **(A)** Left panel—Sanger sequencing, and right panel—fragment analysis. Total RNA was extracted from the conditionally reprogrammed nasal epithelial cells of CF individual with F508del/L88X genotype. RT-PCRs were performed using CFTR-specific primers to amplify L88X and F508del regions. Area under the peak was used to determine expression of L88X transcript compared to F508del. **(B)** RNA-seq of the primary human nasal epithelial cells of healthy and L88X/F508del individuals. Density profile of all expressed genes (top), and relative transcript counts of L88X compared to F508del (bottom). **(C)** Schematic of CFTR-Expression Minigene with abridged introns 1, 2, 3, 4 and 5 constructed in pcDNA5FRT plasmid. *CFTR* exons are shown in boxes and abridged introns in dashed lines. The location of each studied variant is shown relative to the *CFTR* exons and regions predicted to elicit NMD. **(D)** Graph shows relative steady state levels of *CFTR* transcript in HEK293 stable cells expressing wild-type EMG or EMGs with truncations at residue position, as indicated on the labels. Values were normalized to B2M. Mean ± SEM (*n* = 3) measured in triplicates. *P* value was determined by one way ANOVA. No significant difference (n.s.) (*P*>0.01) when compared with *CFTR* mRNA abundance in cells expressing WT-EMG. **(E)** Immunoblot of the naturally occurring 5’-truncations on the steady state amounts of CFTR protein expressed in HEK293 cells. CFTR was visualized with anti-CFTR antibody-596 (CFFT), and anti-Na^+^K^+^ATPase served as control. **(F)** CFTR function measured in CFBE stable expressing L88X. Cells were incubated for 24 h with readthrough compound (G418, 5 μM and 25 μM)/ corrector (lumacaftor, 3 μM) or both. Short-circuit (I_sc_) tracing of CFTR function observed in CFBE-stable cells expressing L88X mounted on Ussing chamber. Change in Isc (ΔI_sc_) was defined as the current inhibited by Inh-172 after sustained Isc responses achieved upon stimulation with forskolin alone or sequentially with ivacaftor. Data are presented as mean ± SEM (n = 3). *P* value was determined by one way ANOVA. **** (*P* ≤0.0001), and *** (*P* ≤0.001) indicate significant difference when compared with forskolin stimulated CFTR function in DMSO (vehicle) treated cells. WT-CFTR function represents forskolin stimulated Isc without modulator treatment in cells expressing EMG i1-i5.

Lastly, we determined what treatment options are appropriate for nonsense variants occurring in the 5’ region of CFTR. This area was reasonable to study as it has been reported that variants that introduce PTCs in the 5’ of processed mRNA may evade NMD [57]. Under these circumstances, translation may initiate at downstream Met codons leading to the synthesis of N-terminal truncated protein. To establish whether 5’ nonsense variants in CFTR evade NMD, we quantified RNA transcripts from primary nasal epithelial cells of a CF individual harboring L88X and the F508del variant. Three methods established that CFTR transcript bearing the L88X variant was stable and at quantities similar to RNA transcripts containing F508del (**Fig 5A and 5B**). Sanger sequencing revealed that transcript bearing the G nucleotide at nt 263 (corresponding to L88X) was almost as abundant as T nucleotide present in transcripts bearing F508del (**Fig 5A**, left panel). Fragment size analysis capitalizing on the 3bp deletion caused by the F508del variant revealed that 233 bp fragments amplified from F508del transcript were of near equal abundance to 236 bp fragments derived from L88X transcript (F508del (52%) and L88X (48%); **Fig 5A**, right panel). As the prior two methods use PCR that may not linearly amplify transcript [58], we performed RNA sequencing of the L88X bearing primary cells. Sequencing depth distribution of all transcribed genes were similar in the F508del/L88X and healthy control nasal cells (**Fig 5B**, left graph). Expression levels of the target gene (*CFTR*), three housekeeping genes (*TBP*, *GAPDH*, and *B2M*), and NMD regulator genes in F508del/L88X sample were in the same range as in healthy control, indicating that NMD machinery was not compromised in the affected individual (**S7A Fig**). The counts of L88X transcripts (n = 7) sequenced from L88X/F508del primary cells were similar to F508del (n = 8; **Fig 5B**, right graph). Exon skipping was not observed in the nasal cells of the individual harboring L88X/F508del (**S7B and S7C Fig**). Since F508del transcript is found at 83% of WT levels [31, 54], we conclude that L88X does not elicit NMD. Absence of NMD was also detected in primary nasal cells of CF individual harboring another 5’ nonsense variant G27X *in trans* with F508del (**S8 Fig).**

Next, we investigated whether other nonsense variants in the 5’ region of *CFTR* evade NMD. Seven different naturally occurring nonsense variants including G27X and L88X were introduced into WT-CFTR EMG i1-i5 (**Fig 5C**). WT-EMG i1-i5 resulted in normal splicing when expressed in HEK293 cells (**S9 Fig**). Each EMG including WT was stably integrated into HEK293 cells and CFTR mRNA abundance was quantified using qRT-PCR. The levels of CFTR RNA transcripts in the cell line bearing the G27X and L88X EMGs were not different from cells with the WT EMG (**Fig 5D**). This result suggested that G27X and L88X transcripts expressed from the CFTR EMG in the HEK293 cells evaded NMD, as observed in the primary cells. Furthermore, CFTR transcript levels in HEK293 stable cell lines expressing the five other 5’ nonsense variants were no different than WT-EMG. The ability of the N-terminus nonsense variants to bypass NMD is likely due to re-initiation of translation downstream start codon(s) [57, 59]. Methionines at codon positions 150,152 and 265 in exons 3, 4, and 7 of CFTR have been shown to be able to operate as alternative start sites in CFTR [60, 61]. IB analysis showed that each of the seven cell lines expressing 5 ‘nonsense variants (lanes 2–6, **Fig 5E, and S10A Fig**) generated two CFTR-specific products ((~135 kDa and ~130 kDa; indicated with stars) consistent with downstream translation initiation. Deglycosylation assay revealed that shortened protein fragments generated from 5’ nonsense variant, e.g. G27X, are immature core glycosylated (**S10B Fig)**. Of note, these proteins are distinct from the molecular mass of immature core-glycosylated protein generated from wild-type-CFTR-EMG (lane 1), Phe508del cDNA (lane 7), and wild-type-CFTR cDNA (lane 9, **Fig 5E**). Thus, 5’ nonsense variants were classified into group B based on CFTR mRNA and protein characteristics. Similar sized molecular mass bands were previously reported in association with nonsense variant Y122X, and 5’ PTC caused by a frameshift variant (c.120del23) when expressed using intronless constructs (i.e. cDNA) [61, 62].

Since mRNA transcripts bearing 5’ nonsense variants were stable, we evaluated the feasibility of readthrough therapy. CFBE stable cells expressing CFTR EMGi1-i5 with L88X were created to test whether readthrough compound (G418) is effective in improving CFTR function. L88X-CFTR generated minimal chloride current (1.18 ± 0.1 μA/cm^2^; **Fig 5F**). Ivacaftor either alone or in combination with lumacaftor was not effective in restoring L88X-CFTR function (**Fig 5F**). Additionally, treatment with G418 at low (5 μM) and high (25 μM) concentrations followed by acute treatment with ivacaftor did not improve L88X-CFTR function. However, G418 in combination with lumacaftor increased activity of L88X-CFTR by ~ 4 fold (3.5% of WT-CFTR function) at 5 μM and by ~ 9 fold (8.5% of WT-CFTR function) at 25 μM concentration (**Fig 5F**, graph).

## Discussion

Genetic variants that generate premature termination codons (PTCs) usually cause severe reduction in protein quantity, either due to nonsense mediated RNA decay (NMD) and/or degradation of any truncated protein that is synthesized [15, 63–65]. We show here that exceptions exist to both paradigms which leads to a reconsideration of variants that might be amenable to protein-targeted therapies. Systematic analysis of variants clustered in four regions of *CFTR* provides compelling evidence that a fraction of PTC-generating variants allow production of protein which can be processed to a stable mature glycosylated form. Importantly, chloride channel function of these mature forms of CFTR can be augmented by FDA approved modulators. Additionally, our results inform where evolving therapeutic approaches might be most effectively employed. For example, compounds that modestly inhibit NMD could be utilized at non-toxic doses to increase the amount of CFTR truncated beyond ICL6 and modulators could be used to achieve therapeutic level of chloride transport. Conversely, *CFTR* transcripts bearing 5’ PTC-generating variants that allow normal levels of RNA transcript would be ideal targets for read-through strategies. Our studies emphasize that the consequences of PTC-generating variants upon RNA and protein can be assembled into groups (**Table 1**), each necessitating different strategies to achieve optimal precision therapy.

**Table 1:** Heterogeneous effects of PTC generating CFTR variants.

Class of PTC generating variants	mRNA abundance/ processing	Protein processing	Protein Function (% WT)	CFTR Modulator Response Expected	Examples
Group A	Reduced	Immature	< 1%	No	R1158X, R1162X, E1371X, Q1382X, Q1390X
Group B	Normal	Immature	< 1%	Yes, but only in combination with translational readthrough drug	E1401X, E1411X, Q1412X, G27X, Q39X, W57X, E60X, L88X, E92X, Y122X
Group C	Normal	Mature	> 1%	Yes	E1418X, E1418RfsX14, S1435RfsX14, S1455X, Q1476X
Group D	Reduced	Mature	> 1%	Yes. NMD inhibition has synergistic effect.	Y1182X, Q1186X, S1196X, W1204X, S1206X
Group E	Aberrant splicing	Mature	> 1%	Yes.	E831X

• Data were collected from intron containing minigenes harboring respective PTC variant expressed in three different cell lines (HEK293, CFBE, and MDCK); and patient-derived conditionally reprogrammed primary nasal epithelial cells

• CFTR modulator refers to FDA approved correctors (lumacaftor and tezacaftor) and potentiator (ivacaftor)

• G418 was the translational readthrough drug tested on CFBE cells stably expressing L88X

• NMD inhibition was achieved by UPF1 siRNA on CFBE cells stably expressing S1196X and R1158X

Our rationale for the selection of nonsense variants from the C-terminal and ICL-6 regions was based on prior evidence that CFTR is stable after truncation in these regions [52, 53]. However, these studies employed complementary DNA (cDNA) constructs that lacked introns and did not undergo pre-mRNA splicing, a requirement for engagement of NMD [27, 28, 65]. Consequently, the cDNA-based studies did not model the *in vivo* effects of the nonsense variants in each region. Assessing the clinical consequences of the PTC-generating variants necessitated that the mRNA be derived from intron-containing constructs. The EMG system employed here faithfully replicates *in vivo* splicing events and engages NMD [33]. A key additional advantage of the EMGs is that variant effect upon mRNA stability and protein synthesis can be evaluated simultaneously [31, 32]. Furthermore, use of the CMV, a potent constitutively active promoter, enables detection and characterization of proteins that are present at low levels *in vivo*. Such studies can provide justification for therapeutic strategies to augment the level and function of truncated proteins generated from genes bearing PTC-generating variants. Furthermore, EMGs provide a viable alternative to primary cell analysis when affected tissues are difficult to procure. This includes variants that are carried by small numbers of geographically dispersed individuals or cell types that cannot be easily accessed (i.e. lung or pancreas). EMGs also allow interrogation of the effects of variants upon CFTR processing and function individually, rather than in a primary cell context where contributions of variants in both *CFTR* genes usually have to be taken into account. Finally, EMGs can be expressed in different cell lines to address the issue of cell-type specific factors. In this study, we employed three cell lines, of which two were of human origin. CFBE cells provide a native human context for *CFTR* expression [47] whereas MDCK cells are of mammalian non-human origin but retain epithelial cell machinery for protein trafficking and polarization [42–48]. HEK293 stable cell lines are useful for rapid evaluation of mRNA stability and protein processing in a human-derived cell model system that does not polarize [66, 67]. Although heterologous expression systems may not capture features of airway cells from primary nasal or bronchial cultures such as assessment of nasal mucociliary clearance [68], they have been deemed sufficient to approve clinical expansion of drug labels by the FDA [69, 70]. Consistent results among the different cell lines verified by observations in available primary cells increase confidence that the observed functional effects are due to heterologous expression of mutant forms of CFTR.

Investigation of nonsense or frameshift variants located in the 3’ region provided an excellent opportunity to test the feasibility of targeting truncated CFTR, as PTC-generating variants located either in the last exon or < 50 nt from the EJC in the penultimate exon are not subject to NMD [17, 34, 65]. As expected, *CFTR* transcripts bearing 8 PTC (6 nonsense and 2 frameshift) variants located in the aforementioned regions were stable, whereas those bearing 3 nonsense variants located elsewhere (Q1390X, Q1382X, and E1371X) were unstable. Synthetic truncations of CFTR established that the C-terminal domain modulates the biogenesis and maturation of CFTR and suggested that variants that introduced PTCs upstream of codon 1390 or downstream of codon 1440 may result in truncated but stable forms of CFTR protein [36, 71]. However, by using EMGs we show that nonsense variants at or upstream of codon 1390 do not generate stable truncated protein due to NMD of mRNA transcript. Consequently, therapeutic targeting of PTC-generating variants upstream of codon 1390 should focus on abrogation of NMD and targeting of truncated CFTR. Conversely, we found that truncations caused by naturally-occurring PTC-generating variants up to codon 1418 were associated with low to wild-type amounts of mature truncated CFTR protein that were partially functional. Consistent with our observation, CFTR bearing E1418X has been reported to be functional in primary nasal epithelial cells derived from an individual with CF [72]. CFTR bearing each of the 5 naturally-occurring PTC variants from 1418 to 1476 were responsive to lumacaftor, tezacaftor, or both in combination, consistent with our observation that correctors increase the level of mature truncated forms of CFTR. Together, our results indicate that PTC-generating variants in the C-terminus of proteins should be carefully evaluated as they may allow generation of stable mRNA and mature truncated protein with residual function. In the case of CF, individuals carrying PTC-generating variants at or downstream of codon 1418 may benefit from protein modulator treatments.

A cluster of 7 naturally occurring CF-causing nonsense variants within exon 22 encoding the ICL6 region were studied as it has been shown that CFTR missing the second nucleotide binding domain (NBD2) and thereafter matures well to form a functional chloride channel at the cell surface [52, 53]. These studies demonstrated that CFTR truncated at codon 1218 (just prior to NBD2) generated protein kinase A (PKA) stimulated halide conductance when expressed in in BHK cells and chloride conductance by single channel recording when embedded in planar lipid bilayers [52, 53]. Our studies revealed that CFTR truncated up to codon 1182 is glycosylated and partially functional whereas truncation 20 residues further upstream (to codon 1162) results in immature protein with no CFTR function. More recently, truncations studied in ATP-binding cassette transporter, Ste6, of yeast demonstrated distinct metabolic stabilities [73]. L1240X and R1268X truncations exhibited similar stabilities as the wild-type protein. In contrast, truncations in between these two locations destabilized the protein emphasizing the fidelity with which Endoplasmic-Reticulum-associated degradation substrates are selected [73].

The truncated forms of CFTR that retained residual chloride channel function, especially Y1182X, were remarkably responsive to ivacaftor when combined with a corrector. We emphasize that modulator mediated improvements in CFTR function observed here in stable cell lines expressing nonsense variants cannot be extrapolated to improvements expected in individuals harboring nonsense variants unless measures are taken to antagonize NMD [74]. Indeed, primary cells demonstrated that CFTR mRNA bearing R1158X and S1196X undergo NMD, and it is reasonable to predict that transcripts *in vivo* bearing each of the remaining 5 nonsense variants in this region would be similarly degraded. We show in cell lines that increasing the stability of transcripts bearing S1196X expressed in CFBE cells by NMD disruption resulted in higher forskolin-activated CFTR chloride currents that were substantially augmented by modulators. However, it remains to be determined whether NMD can be effectively inhibited *in vivo* to stabilize disease-causing PTC transcripts with minimal deleterious impact on the normal transcriptome. In addition to its role in RNA surveillance, NMD is a post-transcriptional regulatory pathway that keeps transcriptome under control from ‘noisy’ expression of faulty transcripts across various mammalian species [75–78]. Therefore, therapeutic strategies based on interference of this pathway are limited. Recently, antisense oligonucleotide mediated reduction of NMD factors have been proposed to be effective and safe to stabilize nonsense transcripts [79]. From a therapeutic perspective, the risks inherent in antagonizing NMD may be better justified when the target RNA transcript encodes a protein that is partially functional and responsive to modulators (e.g. exon 22 nonsense variants).

The importance of understanding pathologic mechanism for treatment of individuals bearing PTC variants is further illustrated by the E831X variant. Hinzpeter and colleagues demonstrated that the nucleotide change that predicted a nonsense variant at codon 831 actually altered RNA splicing, leading to production of minimally functional CFTR missing a single amino acid at codon 831 (del831) [80]. We now show that ivacaftor and correctors augmented current generated by a CFBE cell line stably expressing the E831X-EMG. The most likely target for the modulators is the CFTR isoform missing the amino acid at 831 as it achieves mature glycosylation and is partially functional [80]. We also show that correctors augment potentiator response in primary nasal cells with genotype E831X/F508del. Based on drug response data from stable cells that had hemizygous expression of F508del-CFTR, we inferred that increased response of modulators in primary nasal cells was due to their action on E831X allele rather than F508del alone. In support of this supposition, a recent study has confirmed that corrector-potentiator combination therapy is beneficial to CF individuals with the F508del/E831X genotype [13] and the FDA has now approved use of ivacaftor for E831X based on analysis of *in vitro* data [81].

*CFTR* mRNA transcripts bearing nonsense variants in the 5’ exons did not elicit NMD thereby providing an opportunity for protein synthesis. Our observation is consistent with studies of other genes where mRNAs with nonsense and frameshift variants in the first exon do not engage NMD due to internal methionine usage [57, 61, 82–85], even though mechanistic models predict that variants should activate NMD [17, 86]. It is postulated that translation from downstream Met codons removes complexes that occur at exon-exon junctions from pioneer transcripts and in doing so, eliminates the trigger for NMD [59]. Here, we show in both primary and stable cells that five disease-causing nonsense variants located up to exon 4 of CFTR resist NMD. We propose that NMD was not engaged due to removal of complexes since each of the five nonsense variants in 5’ exons produced protein of a molecular weight consistent with translation initiation from internal methionine at codon 254. RNA sequencing provided evidence that NMD machinery was not compromised in the primary nasal cells of an individual with one of the 5’ nonsense variants. The proteins generated from internal methionines had residual CFTR chloride channel activity, as previously reported [60], but were poorly responsive to CFTR modulators.

From a treatment perspective, 5’ nonsense variants that do not affect mRNA stability due to internal translation initiation are attractive targets for readthrough therapeutics. However, incorporation of a foreign amino acid at a premature-termination codon may not be sufficient to restore protein function, if the intended residue at this position is critical [87]. Therefore, readthrough therapeutics in combination with treatment aimed to increase protein stability and function, e.g. CFTR directed modulators (correctors and/or potentiators), should be efficacious for treating PTCs with stable mRNA. G418, a neomycin analog, is the most widely used compound for readthrough of PTCs [88–90]. Our detailed studies of L88X in its native CFTR context in primary cells and in CFTR expression minigene in CFBE cells showed that G418 in combination with lumacaftor significantly increased CFTR function of L88X. Similarly, other investigators have shown improvement in CFTR function by G418-CFTR corrector and potentiator co-treatment in intestinal organoids from CF individuals harboring E60X (exon 3) [91]; and Fisher Rat Thyroid (FRT) or HEK293 cells expressing Y122X (exon 4) [62, 92]. Although the former study did not measure RNA levels in intestinal organoids, and the latter studies utilized hybrid minigenes or cDNA constructs that could not evoke NMD, it can be predicted from our EMG results that E60X and Y122X transcripts are stable *in vivo* and therefore likely to be responsive to readthrough agents. Interestingly, there is mounting evidence that efficacy of PTC suppression by readthrough compounds is affected by the sequence context of nonsense variants [93, 94]. Of note, the three 5’ nonsense variants (E60X, L88X, and Y122X) that show improvement in CFTR function by G418-modulator co-therapy have different sequences coding for nonsense variants (UAG, UGA, and UAA), different flanking amino acids (ArgXGlu, PheXTyr, and IleXLeu), and different nucleotide sequences at -1 (A,U, and U), and +4 positions (C,U, and C) [62, 91]. There could be numerous factors that affect efficiency of readthrough, but data presented here and by others [62, 91, 92, 94] show that favorable clinical outcome is possible using readthrough and protein modulator co-therapies for those 5’- nonsense variants that unequivocally produce normal mRNA levels.

In summary, our systematic approach reveals that PTC-generating variants have a variety of consequences that can be exploited for therapeutic purposes. We show that the location of PTC-generating variants can help predict whether NMD may be engaged but that effects on protein stability and residual function are less obvious. Three scenarios are evident that necessitate different strategies. First, individuals harboring disease-causing PTC-generating variants that produce stable RNA and mature protein (e.g. 3’ end) are eligible for currently available protein modulators without a need for NMD inhibitor although translational read-through drugs might be beneficial. Second, individuals harboring variants generating unstable mRNA but mature protein (e.g. exon22/ICL6) should be considered for NMD inhibitor and protein modulator therapy. Finally, individuals with nonsense variants (e.g. 5’ end) where mRNA abundance is not affected due to use of alternative start sites should be amenable to read-through and protein modulator treatment without the need for NMD inhibitor. These results show that nonsense and frameshift variants that introduce PTCs can have markedly different effects on CFTR protein synthesis and eligibility for modulator treatments.

## Materials and methods

### Ethics statement

This study was approved by the Institutional Review Boards at Johns Hopkins Medicine, Baltimore, and Case Western Reserve University/University Hospitals Case Medical Center, Cleveland (approval numbers IRB00116966 and UHCMC#10-14-14). Written informed consent was obtained from all subjects.

### Study design

The purpose of this study was to systematically evaluate mRNA stability, protein production, and/or function of PTC-generating variants in CFTR to identify which variants allow generation of CFTR responsive to currently available modulator therapies and those that require alternative therapeutic approaches. Twenty six PTC generating variants were selected. To explore mRNA and protein using a single platform we generated four WT-EMGs. Each variant EMG was created by site directed mutagenesis of WT-EMG. Variant and WT-EMGs were expressed in three different stable cell lines. Primary nasal cells obtained from CF individuals harboring PTC generating variants were conditionally reprogrammed. *CFTR* mRNA abundances, mRNA stability, and splicing were assessed by qRT-PCR, Sanger sequencing, fragment analysis, pyrosequencing, and RNA sequencing. CFTR protein processing was evaluated by immunoblotting and glycosidase digestion. CFTR function was determined by short-circuit current (I_sc)_ measurements on Ussing chambers. To evaluate whether modulators were effective in improving CFTR function of cells expressing PTC generating variants, following FDA approved small molecules were selected; (i) Correctors (lumacaftor and tezacaftor), and (ii) Potentiator (ivacaftor). CFTR specific function in the cells was calculated as change in Isc (ΔIsc) defined as the difference between the sustained phase of the current response after stimulation with forskolin and the baseline achieved after adding Inh-172. *UPF1* siRNA was used to determine effect of NMD inhibtion on CFTR function in cells expressing EMG harboring nonsense variants that produced unstable mRNA. G418 was used to evaluate whether translational readthough resulted in improvement of CFTR function in cells expressing EMG harboring nonsense variants that produced stable mRNA. Each experiment was repeated at least 3 times.

### Creation of EMG constructs

Four EMGs were created as described previously [7, 32, 33]. CFTR-EMG-i1-i5 contained: abridged intron 1 (216 bp of 5' and 212 bp of 3'), abridged intron 2 (311 bp of 5' and 264 bp of 3'), abridged intron 3 (374 bp of 5' and 456 bp of 3'), abridged intron 4 (307 bp of 5' and 333 bp of 3'), and full-length intron 5 (882 bp). CFTR-EMG-i14-i18 contained: full-length intron 14 (2272 bp), abridged intron 15 (259 bp of 5' and 359 bp of 3'), full-length intron 16 (668 bp), abridged intron 17 (330 bp of 5' and 302 bp of 3'), and abridged intron 18 (333 bp of 5' and 339 bp of 3'). CFTR-EMG-i21-i22 contained abridged intron 21 (227 bp of 5' and 222 bp of 3'), and abridged intron 22 (191 bp of 5' and 256 bp of 3'). CFTR-EMG-i25-i26 contained full-length intron 25 (598 bp) and full-length intron 26 (1343 bp). A single nucleotide alteration c.3519T>G (p.Gly1173Gly) was introduced to avoid missplicing of EMG-i21-22.

### Generation of stable cell lines

Human embryonic Kidney (HEK293), CF bronchial epithelial (CFBE41o-), and Madin Darby Canine Kidney (MDCK II) cells each containing a Flp Recombinase Target (FRT) integration site, which facilitates site-specific recombination, were used to create stable cell lines expressing WT-CFTR-EMG or variant CFTR-EMG, as described previously [32, 46, 47, 49, 50].

### Collection of primary nasal cells

Nasal cells were collected from CF and healthy individuals following IRB protocols at Johns Hopkins University, Baltimore (IRB# 00116966) and Case Western Reserve University, Cleveland (IRB# UHCMC#10-14-14). An experienced physician performed endoscopic procedures to harvest nasal cells from individuals after informed consent was obtained. Nasal epithelial cells were collected from the mid-part of the inferior turbinate of healthy/CF individuals by brushing with interdental brushes, after spraying a topical anesthetic on the nasal mucosa.

### Isolation, expansion, and culture of primary human nasal epithelial cells

Primary human nasal epithelial (HNE) cells were harvested from CF individuals and healthy volunteers. Expansion and culture of nasal epithelia were performed as previously described [95, 96]. Briefly, nasal cells were expanded by culturing in DMEM/F-12 media in the presence of 10 μM Y-27632, a ROCK inhibitor, and irradiated fibroblast feeder cells. After 2 passages of expansion, cells were seeded (5x10^5^ cells/cm^2^) onto snap-well inserts (Costar #3801). On confluence (day 5–7), propagation media was replaced with differentiation media containing Ultroser G serum substitute (Pall; Port Washington, NY) without reagent Y. The following day, cells were maintained at an air-liquid interface (ALI) by removing media from the apical compartment and providing media to the basal compartment only. The apical surface was washed with phosphate-buffered saline (PBS) to remove any mucus accumulation, and the medium was replaced in the basal compartment every 48 h. Cells were maintained at 37°C and 5% CO2.

### Real time quantitative RT-PCR

CFTR mRNA abundance in stable cells was determined by real-time, quantitative reverse transcriptase polymerase chain reaction (qRT-PCR). Briefly, cDNAs were synthesized using iscript cDNA synthesis kit (Biorad#170–8890). PCRs for target gene (CFTR) and housekeeping gene (B2M) were performed using SsoAdanced Universal SYBR Green mix (Biorad#172–5271) Sequence of CFTR primer pair was: CFTR, forward 5’-TGACCTTCTGCCTCTTACCA-3’, reverse 5’-CACTATCACTGGCACTGTTGC-3’. B2M primer pairs are commercially available from Biorad (#qHsaCID0015347). Real time qRT-PCR data were obtained on CFX connect Real time system (BioRad). Expression levels were calculated by subtracting housekeeping control (B2M) cycle threshold (Ct) values from target (CFTR) Ct values to normalize for total input, resulting in ΔCt levels. Relative transcript abundance was computed as 2^−ΔCt. Each sample was run in triplicate.

### Analysis of alternate CFTR transcripts in primary nasal cells

Since CF individuals harboring nonsense variant were in compound heterozygosity with a different CFTR variant, we were able to quantify relative abundance of each allele. Reverse transcription (RT) was carried out using 50–250 ng total RNA using i-Script cDNA synthesis kit (BioRad#170–8890). The reaction mix was incubated for 5 min at 25°C, 30 min at 42°C and 5 min at 85°C. Undiluted cDNA product was used to perform following assays.

#### Fragment analysis

RT-PCR was performed using 2 μl cDNA in a standard 50 μl reaction set up containing: 2X “KOD hot start" master mix, and 10 μM each forward and reverse primers. The forward primer was fluorescently-labeled with 6—FAM (carboxyfluorescein) at the 5' end. PCR conditions were 2 min at 95°C, followed by 35 cycles of 20 sec at 95°C, 10 sec at the annealing temperature, and 15 sec at 70°C. The RT-PCR products were mixed with Hi-Di Formamide (Applied Biosystems) and an internal size standard (GeneScan -500 Rox, Applied Biosystems). Products were separated by capillary electrophoreses on an ABI 3100 Genetic Analyser using POP4 polymer (Applied Biosystems) and analyzed with the Gene Mapper Software Version 3.7 (Applied Biosystems). Primer sequences were CFTR-e11-Forward: /56-FAM/ATT TCA TTC TGT TCT CAG TT, CFTR-e13-Reverse: 5’- TCA GCA TCT TTG TAT ACT GC-3’.

Automated sizing of DNA fragment was performed by the electrophoresis of RT-PCR product on Fragment Analyzer Automated CE System using 35 bp-1500 bp size standards available from Advanced Analytical Technologies.

#### Pyrosequencing

To determine relative expression of alternate allele’s pyrosequencing was performed [97]. Both PCR and sequencing primers were designed using PyroMark Assay Design Software. Reverse PCR primer was biotin-labeled to enable mobilization of streptavidin-coated beads. The RT-PCR was performed using 2 μl cDNA in a standard 50 μl reaction set up containing: PyroMark PCR master mix (2X) 25 ul, and Primer mix (10X) 5 ul, following manufacturer’s instructions (PyroMark PCR kit (Qiagen#978703). Coral Load concentrate provided in the kit was avoided. PCR conditions were 2 min at 95°C, followed by 40 cycles of 30 sec at 94°C, 30 sec at 60°C, 30 sec at 72°C, and final extension of 10 min at 72°C. The products were sequenced according to PyroMark Q24 system (Qiagen) with 0.4 μM of specific pyrosequencing primers and pyrograms were analysed with the software PyroMark Q24 V.2.0.6 (Qiagen).

#### RNA sequencing

Using 1.0 μg of total RNA isolated from conditionally reprogrammed primary nasal epithelial cells, library preparation and RNA-Seq were performed at the Johns Hopkins Medical Institutions Deep Sequencing and Microarray Core Facility. RNA-Seq library was constructed using the TruSEQ RNA Sample Prep Kit v2 (Illumina, San Diego, CA, USA), and 50 million paired end reads were obtained from 75 cycle run on Illumina Next Seq 500 platform. Raw reads were aligned to the reference genome (hg19) using the Bowtie2 algorithm [98], and splice junctions were identified via Tophat2 (v2.0.13) [99] from the Tuxedo software suite. CuffQuant and Cuffdiff (Cufflinks v2.2.1) [100] were then used to assemble transcripts, estimate allele counts in compound heterozygous sample, and test for differential expression of CFTR, nonsense mediated decay (NMD) regulator genes, and housekeeping genes between CF and healthy nasal epithelial cells. To quantitatively visualize the splice junctions of *CFTR*, sashimi plots were generated from RNA sequencing data using the Integrative Genomics Viewer.

### Assessment of CFTR function by short-circuit current measurement

#### CFBE stable cells

Cell counting was performed using Countess II Automated Cell Counter (ThermoFisher Scientific). About 2.5-4x10^5^ cells were plated onto Snapwell filters (12 mm filter diameter with 0.4 μm pore diameter; Corning Costar #3407) with daily feeding from both apical and basolateral sides. Transpithelial resistance was measured every day using Epithelial Voltohmmeter (EVOM, World Precision Instrument). When resistance approached ~ 180–200 Ω in roughly 5 days, cells were incubated with correctors (lumacaftor/tezacaftor or both, 3 μM each) or readthrough compound (G418, 5 μM and 25 μM). Although in few cell lines maximum resistance observed was 150 Ω. After one or two days of drug treatments, filters were mounted into Ussing chambers and short circuit currents (I_sc_) were measured with a VCC MC6 or VCC MC8 multichannel voltage-current clamp amplifier (Physiologic Instruments). Asymmetric apical and basolateral buffers were used to create a chloride gradient, with the apical buffer composed of 145 mM NaGluconate, 1.2 mM MgCl_2_, 1.2 mM CaCl_2_, 10 mM dextrose, 10 mM HEPES, and the basolateral buffer composed of 145 mM NaCl, 1.2 mM MgCl_2_, 1.2 mM CaCl_2_, 10 mM dextrose, 10 mM HEPES. Buffers were maintained at 37°C and air was bubbled in to introduce circulation. After stabilization of transepithelial current, 10 μM forskolin (Selleckchem) was added to the basolateral chamber to stimulate generation of cAMP and activation of CFTR, followed by administration of 10 μM CFTR inhibitor-172 (Selleckchem) in the apical chamber to block CFTR-mediated currents. Data were acquired with the software Acquire and Analyze (Physiologic Instruments). CFTR specific function in the cells was calculated as change in Isc (ΔIsc) defined as the difference between the sustained phase of the current response after stimulation with forskolin and the baseline achieved after adding Inh-172.

#### MDCK stable cells

Protocol was similar to assessment of CFTR function in CFBE stable cells except 1x10^5^ cells were plated, resistance peaked and then dropped after ~ 5–6 days, cells were incubated with modulators on ~ day 3 when resistance was ~250–300 Ω, and measurement of short circuit currents (I_sc_) was performed on ~ day 5.

#### Primary nasal cells

Apical and basolateral chambers contained the same bathing solution containing (mM): 115 NaCl, 25 NaHCO3, 5 KCl, 2.5 Na2HPO4, 1.8 CaCl2, 1 MgSO42, and 10 dextrose (pH = 7.4). The bath was maintained at 37°C, and continuously circulated by carbogen gas lift (95% O_2_/5% CO_2_). After stabilization of baseline, following inhibitors and activators of Isc were sequentially added: sodium (Na+)-channel blocker Amiloride (100 μM) to inhibit apical epithelial Na+ channel (ENaC); cAMP agonists Forskolin (10 μM) and 3-isobutyl-1-methylxanthine (IBMX 100 μM) to activate the transepithelial cAMP-dependent current (including Cl− transport through CFTR channels); and CFTR inhibitor CFTRinh172 (10 μM) to specifically inhibit CFTR. Data were acquired with the software Acquire and Analyze version 2.3.159 (Physiologic Instruments). CFTR specific function in the cells was calculated as change in Isc (ΔIsc) defined as the difference between the sustained phase of the current response after stimulation with forskolin and the baseline achieved after adding Inh-172.

### Statistical analysis

Statistical analysis was performed, and graphs were generated using GraphPad Prism6 (GraphPad Software Inc.). Results are presented as means ± SEM, with the number of experiments indicated. One-way ANOVA followed by Dunnett's multiple comparisons test was performed. *P* values ≤ 0.05 were considered significant. Individual-level data underlying each graph and exact *P* values are provided in S1–S5 Data.

## Supporting information

S1 Text (DOCX)

S1 Fig Fragment analysis of the RT-PCR of the total RNA extracted from HEK293 stable cells expressing wild-type EMG-i25-26 (related to Fig 1). Inset shows agarose gel electrophoresis. Plasmid harboring intronless full-length CFTR was used a positive control. Samples with no RT, water control, and parental cells that lack endogenous CFTR expression were used as negative controls. Automated sizing of DNA fragment was performed by the electrophoresis of RT-PCR product on Fragment Analyzer Automated CE System using 35 bp-1500 bp size standards available from Advanced Analytical Technologies. UM indicates upper marker and LM indicates lower marker. RFU refers to Relative Fluorescence Units.
(PPTX)

S2 Fig IB showing sensitivity of CFTR to PNGase F and Endo H (related to Fig 1). Mature complex glycosylated band is sensitive to PNGase F only, whereas immature core glycosylated band is sensitive to both PNGase F and Endo H. Whole lysates were collected from HEK293 cells expressing WT-EMG or EMGs with different PTC-generating variants. Deglycosyation was achieved by Endo H and PNGase F following manufacturer’s protocol (New England Biolabs), except that denaturation was performed at 37°C. Fifty microgram of total cell lysate was used for deglycosylation followed by electrophoresis. Respective undigested lysates (30 μg) were used as controls. Lysates from cells expressing either intronless WT-CFTR or F508del served as additional controls. IB was probed with anti-CFTR antibody (596 # Cystic Fibrosis Foundation Therapeutics). Arrows indicate mature and immature forms of either full-length or truncated CFTR. Both light and dark exposures are provided.
(PDF)

S3 Fig Fragment analysis of the RT-PCR of the total RNA extracted from HEK293 stable cells expressing wild-type EMG-i21-22 (related to Fig 2). Inset shows agarose gel electrophoresis. A single nucleotide alteration c.3519T>G (p.Gly1173Gly) was introduced to avoid missplicing of EMG-i21-22. Plasmid harboring intronless full-length CFTR was used a positive control. Samples with no RT, water control, and parental cells that lack endogenous CFTR expression were used as negative controls. Automated sizing of DNA fragment was performed by the electrophoresis of RT-PCR product on Fragment Analyzer Automated CE System using 35 bp-1500 bp size standards available from Advanced Analytical Technologies. UM indicates upper marker and LM indicates lower marker. RFU refers to Relative Fluorescence Units.
(PPTX)

S4 Fig Representative IB showing sensitivity of CFTR to PNGase F and Endo H (related to Fig 2). Mature complex glycosylated band is sensitive to PNGase F only, whereas immature core glycosylated band is sensitive to both PNGase F and Endo H. IB was probed with anti-CFTR antibody-MM13-4 (EMD Millipore).
(PPTX)

S5 Fig Fragment analysis of the RT-PCR of the total RNA extracted from HEK293 stable cells expressing wild-type EMG-i14-18 (related to Fig 4). Inset shows agarose gel electrophoresis. Plasmid harboring intronless full-length CFTR was used a positive control. Samples with no RT, water control, and parental cells that lack endogenous CFTR expression were used as negative controls. Automated sizing of DNA fragment was performed by the electrophoresis of RT-PCR product on Fragment Analyzer Automated CE System using 35 bp-1500 bp size standards available from Advanced Analytical Technologies. UM indicates upper marker and LM indicates lower marker. RFU refers to Relative Fluorescence Units.
(PPTX)

S6 Fig Sanger sequences of splice isoforms produced by E831X variant (related to Fig 4). Total RNA was isolated from HEK293 cells stably expressing EMG-i14-18-E831X. RT-PCR was performed using CFTR specific primers.
(PPTX)

S7 Fig RNA-seq analysis of primary nasal epithelial cells of individual with *CFTR* genotype L88X/F508del (related to Fig 5). **(A)** Heat map showing relative expression of *CFTR* and genes implicated in NMD. Housekeeping genes (*B2M*, GAPDH, and *TBP*) are shown as controls. **(B)** Sashimi plots showing exon 3 harboring L88X variant is normal spliced. Per-base expression is plotted on y-axis of Sashimi plot, genomic coordinates on x-axis, and spliced mRNA are shown on bottom (exons in black, introns as lines with arrow heads). RNA from healthy individual was used as control. **(C)** Splicing patterns in *B2M* from both L88X/F508del and healthy individual are shown as controls.
(PPTX)

S8 Fig Sanger sequence of the RT-PCR product obtained from the primary nasal epithelial cells of individual with CFTR genotype G27X/F508del (related to Fig 5). Illustration on the top shows location of CFTR-G27X variant in the exon 2 indicated by vertical arrow. Horizontal arrows indicate location of CFTR specific forward and reverse primers used in the RT-PCR.
(PPTX)

S9 Fig Fragment analysis of the RT-PCR of the total RNA extracted from HEK293 stable cells expressing wild-type EMG-i1-i5 (related to Fig 5). Inset shows agarose gel electrophoresis. Plasmid harboring intronless full-length CFTR was used a positive control. Samples with no RT, water control, and parental cells that lack endogenous CFTR expression were used as negative controls. Automated sizing of DNA fragment was performed by the electrophoresis of RT-PCR product on Fragment Analyzer Automated CE System using 35 bp-1500 bp size standards available from Advanced Analytical Technologies. UM indicates upper marker and LM indicates lower marker. RFU refers to Relative Fluorescence Units.
(PPTX)

S10 Fig IB showing CFTR protein processing of 5’-nonsense variants (related to Fig 5). (**A)** Immunoblot of the naturally occurring 5’-truncations on the steady state amounts of CFTR protein expressed in HEK293 cells. CFTR was visualized with anti-CFTR antibody-596 (CFFT). **(B)** Representative IB showing sensitivity of CFTR to PNGase F and Endo H. Mature complex glycosylated band is sensitive to PNGase F only, whereas immature core glycosylated band is sensitive to both PNGase F and Endo H. Fifty microgram of total cell lysate was used for deglycosylation followed by electrophoresis. Respective undigested lysates (30 μg) were used as controls. IB was probed with anti-CFTR antibody (596 # Cystic Fibrosis Foundation Therapeutics). Arrow indicates immature form of shortened CFTR produced from EMG i1-i5 harboring G27X.
(PPTX)

S1 Data Raw values used to generate graphs in Fig 1. The raw data presented in worksheet serve as underlying data for Fig 1B, 1G and 1H.
(XLSX)

S2 Data Raw values used to generate graphs in Fig 2. The raw data presented in worksheet serve as underlying data for Fig 2B, 2G and 2H.
(XLSX)

S3 Data Raw values used to generate graphs in Fig 3. The raw data presented in worksheet serve as underlying data for graph in Fig 3B, 3D and 3F.
(XLSX)

S4 Data Raw values used to generate graphs in Fig 4. The raw data presented in worksheet serve as underlying data for graph in Fig 4C.
(XLSX)

S5 Data Raw values used to generate graphs in Fig 5. The raw data presented in worksheet serve as underlying data in Fig 5D and 5F.
(XLSX)
