# Supplementary material for: Capitalizing on the heterogeneous effects of CFTR nonsense and frameshift variants to inform therapeutic strategy for cystic fibrosis
Source: PLoS Genet. 2018 Nov 16;14(11):e1007723. doi: 10.1371/journal.pgen.1007723 (PMC6267994; doi:10.1371/journal.pgen.1007723)
Supplement: S1 Text — (DOCX) [file pgen.1007723.s001.docx]

**SUPPLEMENTARY MATERIAL**

**MATERIALS AND METHODS**

**Creation of expression minigenes**

Four EMGs were created by inserting either abridged intron (containing partial 5’ and 3’intron sequence) or a complete intron into a pcDNA5FRT plasmid harboring full-length CFTR cDNA. CFTR introns were amplified from the genomic DNA of healthy individual. Nine abridged introns were amplified using primer pairs from the respective introns and flanking exons, and five full-length introns were amplified using primers from the exons only (See below). The protocol for inserting an individual intron into a plasmid harboring CFTR cDNA involved amplification, fusion of 5’ and 3’ ends (for abridged intron only), sticky feet mutagenesis, transformation, plasmid extraction, and sequence verification, as described previously [1-3]. This protocol was repeated to incorporate additional introns at the respective locations. A single nucleotide alteration c.3519T>G (p.Gly1173Gly) was introduced in EMG-i21-22 to remove splicing artifact caused by the usage of alternative splice site. Site directed mutagenesis primers employed to create c.3519T>G variant in EMG-i21-i22 were Forward primer: GACATG CCAACAGAAGGGAAACCTACCAAGTCAAC and Reverse primer: GTTGACTTGGTAGGTTTCCCTTCTGTT GGCATGTC

**Primers used for the creation of Expression minigene (EMG) constructs**

**Site directed mutagenesis**

The Quickchange XL II mutagenesis kit (Agilent, Santa Clara, CA, USA) was used with minor modifications to introduce CFTR variants into the pcDNA5FRT plasmid harboring WT-CFTR EMG. The site directed mutagenesis primers to create nonsense and frameshift variants were designed using QuickChange Primer Design tool freely available at <https://www.genomics.agilent.com/primerDesignProgram>. Briefly, the mutagenesis involved: thermal cycling (2X “KOD hot start" master mix, 20 ng wild-type EMG, 125 ng primers), *DpnI* (NEB) digestion of the PCR products, transformation of XL10-Gold ultracompetent cells (Agilent), selection of the colonies on LB-Ampicillin plates (Quality Biologicals). A minimum of six plasmid minipreps were prepared (Denville Spinsmart Plasmid Miniprep DNA Purification Kit). Sanger sequencing of the minipreps was performed to verify mutagenesis at the desired location (Synthesis and Sequencing Facility, Johns Hopkins University School of Medicine). Selected miniprep plasmid was used to transform DH5α competent cells (Invitrogen), and DNA maxipreps were prepared (Qiagen Plasmid Plus Maxi Kit). Entire CFTR-EMG including CMV promoter and polyA was sequenced to confirm the presence of the variant of interest and the absence of nucleotide changes elsewhere.

**Site directed mutagenesis primers**

**Generation of stable cell lines** **with integrated CFTR variants**

Cystic Fibrosis Bronchial Epithelial (CFBE41o-), Human Embryonic Kidney (HEK293), and Madin Darby Canine Kidney (MDCK II) each containing a Flp Recombinase Target (FRT) integration site that facilitated site-specific recombination were used to create stable cells expressing WT-CFTR-EMG or variant CFTR-EMG, as described previously [1, 4, 5]. Each of these cell lines does not have endogenous expression of CFTR at mRNA and protein levels.

*CFBE stable cells* CF bronchial epithelial (CFBE41o-) cells containing a Flp Recombinase Target (FRT) integration site [4] were grown in complete media supplemented with 100 μg/ml Zeocin (Gibco or ThermoFisher). Prior to transfection, cells were seeded in collagen-coated 6-well plates and grown to >70% confluency. Collagen coating was achieved by applying a mixture of 5 ml of 0.1% bovine serum albumin (MilliporeSigma), 500 μl rat tail Collagen I (Life Technologies – 3mg/ml), and 500μl of human fibronectin (Sigma-Adrich – 1mg/mL) diluted in 44 ml MEM to each well, before aspirating the mixture and allowing the plates to dry for at least 1 hour. 0.5 μg of CFTR-EMG plasmid combined with 4.5 μg of pOG44 Flp-recombinase plasmid was transfected using Lipofectamine LTX (ThermoFisher Scientific # 15338100). Cells were incubated for 48 hours and then split 1:4 into collagen coated 6-well plates. Media was changed after 48 hours to include 50 μg/ml Hygromycin B for 1-2 days then changed again with media containing 100 μg/ml Hygromycin B. Cells remained under Hygromycin selection until distinct clones were observed in transfection wells and all cells in mock transfected wells had died. Cells were expanded to uncoated vessels for characterization.

*HEK293 stable cells* Protocol was similar to creation of CFBE stable cells with following differences. Plates were uncoated, 0.4 μg of CFTR-EMG plasmid was combined with 3.6 μg of pOG44 Flp-recombinase plasmid, transfection was done using Lipofectamine2000 (ThermoFisher Scientific # 11668019), and Hygromycin (100 μg/ml) was added at the first day of selection.

*MDCK stable cells* Protocol was similar to creation of HEK293 stable cells except transfection was done using Lipofectamine3000 (ThermoFisher Scientific # L3000015).

*Verification of CFTR EMG integration in stable cell lines* Extraction of total RNA from the hygromycin resistant cells was performed, cDNA was prepared, CFTR was amplified using exon spanning primers, and Sanger sequencing was performed to confirm CFTR integration and splicing.

**RNA Isolation**

*Stable cells* Total cellular RNA was isolated from the cultured cells. Cells were washed with PBS made with DEPC-treated H_2_O and lysed by addition of 500 µl of TRIzol reagent (Life Technologies) followed by centrifugation through a shredder column (Denville). Flow through was collected and 200 µl chloroform was added. Aqueous and organic phases were allowed to separate at room temperature for 5 min followed by centrifugation at 12,000xg for 5 minutes at 4°C. Clear aqueous layer containing RNA was collected, mixed with 70% ethanol, and further purified using the SpinSmart RNA binding columns, according to the manufacturer’s instructions (Denville#CM-610250). The RNA content and purity were determined using NanoDrop (ThermoScientific).

*Primary nasal epithelial cells* Protocol was similar to RNA extraction in stable cells except an additional step of beat beating (Zirconium beads, OPS diagnostics #80025029) using FastPrep-24 (MP) was performed for effective lysis.

**CFTR mRNA stability assay**

HEK293 stable cells expressing WT-EMG or EMG expressing nonsense variant were treated with actinomycin D (5 µg/mL) to inhibit transcription for the indicated times. Total RNA was isolated, cDNA was synthesized, and the transcript abundance was determined by real-time quantitative RT-PCR and normalized to B2M.

**siRNA treatment**

About 2.5-4x10^5^ CFBE stable cells were plated onto Snapwell filters (12 mm filter diameter with 0.4 µm pore diameter; Corning Costar #3407). Cells were transfected with following siRNA on day 3: ON-TARGETplus SMARTpool human UPF1 (Dharmacon, Cat#L-011763-00), ON-TARGETplus Control Pool Non-targeting pool (Dharmacaon, D-001810-10-20), Accell Control pool human GAPDH (Thermo Scientific Dharmacon D-001930-10-20). The siRNA and Lipofectamine RNAiMAX were diluted separately in Opti-MEM (125 µl each), and combined after 5 min incubation at room temperature. The siRNA-RNAiMAX mix was left to incubate for 25 min at room temperature after which the siRNA-RNAiMAX mix (250 µl/Snapwell) was added to the apical compartment of Snapwell filters. Basolateral compartment received Opti-MEM only. After 4 h post-transfection, Dulbecco’s Minimum Essential Media (DMEM) containing 20% FBS without penicillin-streptomycin was added to both apical (250 µl/Snapwell) and basolateral (1.5 ml/well) compartments. Following day (day 1), media was removed from both apical and basolateral sides, and fed with DMEM media containing 10% FBS. On day 2 post-transfection, cells were treated with either DMSO (0.03%) or lumacaftor (3 µM) from both apical and basolateral compartments. On day 4 post-transfection (total 7 days from the day of seeding the cells), snapwells were mounted on Ussing Chambers for short-circuit current measurements.

**Immunoblot analysis**

Total protein was isolated from cells using cOmplete Lysis-N solution (Roche#04719956001) containing protease inhibitor cocktail tablets (Roche#16858101). The lysates were centrifuged and protein concentrations were estimated using the Microplate BCA Protein Assay kit (Thermo Scientific#OF184596) according to the manufacturer’s instructions. The lysates were subjected to immunoblot analysis. Immunoblotting was performed by denaturing 40 μg of protein at 37°C for 30 minutes (CFTR and Na^+^K^+^ATPase), or 100°C for 5 min (other proteins), in Laemmli sample buffer (Biorad) containing 200 mM DTT (CFTR and Na^+^K^+^ATPase) or 5% β-mercaptoethanol (other proteins). Samples were subjected to SDS-polyacrylamide gel electrophoresis using 7.5% (CFTR, Na^+^K^+^ATPase, and UPF1) or 4-20% (other proteins) Mini-PROTEAN® TGX™ precast gels (BioRad, Hercules, CA, USA) and resolved at 140 V over ~ 2 hours. Proteins were transferred to a PVDF membrane (Biorad#BR20160914) membrane in transfer buffer containing 25 mM Tris, pH 8.3, 192 mM glycine, 0.01% SDS, and 15% methanol using a Bio-Rad Trans-blot turbo semidry transfer system (Biorad) set at 2.5 mA/25 V/10 min. Membranes were blocked in 5% nonfat dry milk, Phosphate buffered saline (PBS), and 1% Tween-20 (PBS-T) at room temperature for 1 hour. Membranes were exposed to antibodies that recognized CFTR, UPF1, and GAPDH. Equivalent protein loading between the samples was verified by probing membranes for β-actin or Na^+^K^+^ATPase. Primary antibodies were used at 1:100 to 1:5000 dilutions in PBST or 5% BSA for 1h at room temperature or 18 hours at 4°C. Membranes were exposed to anti-mouse, anti-rabbit, or anti-goat secondary antibodies conjugated with horseradish peroxidase at a dilution of 1:10,000 to 1:150000 in PBS-T for 1 hour at room temperature. Signals were detected with ECL Prime chemiluminescence detection system (GE Healthcare, Pittsburgh, PA, USA), and exposure to x-ray film (GE Healthcare 28-9068-39).

**Antibodies used for immunoblot analysis**

**Glycosidase treatment**

To remove N-glycans, cell lysates were digested with endo H (NEB # P0702S) and PNGase F (NEB # P0704S) following manufacturer’s protocol. Briefly, 50 µg aliquot of cell lysate was taken, added 1 µl of 10 X denaturating buffer, and water to make 10 µl reaction. Denaturation was carried out at 37°C for 30 min instead of heating at 100°C for 10 min to prevent aggregation of CFTR protein. Thereafter, added 2 µl of 10X glycobuffer, 5 µl Endo H and 2 µl PNGase F in the respective tubes, and water to make 30 ul final reaction. Digestion was carried out by incubation at 37°C for 1 h. To avoid inhibition of PNGase, tubes were chilled after denaturation step, and 5 ul of 10% NP-40 was added. The mobility shift of deglycosylated CFTR was visualized by immunoblot analysis.

**REFERENCES**

1. Sharma N, Sosnay PR, Ramalho AS, Douville C, Franca A, Gottschalk LB, et al. Experimental assessment of splicing variants using expression minigenes and comparison with in silico predictions. Hum Mutat. 2014;35(10):1249-1259. doi: 10.1002/humu.22624 [doi].

2. Lee M, Roos P, Sharma N, Atalar M, Evans TA, Pellicore MJ, et al. Systematic Computational Identification of Variants That Activate Exonic and Intronic Cryptic Splice Sites. Am J Hum Genet. 2017;100(5):751-765. doi: 10.1016/j.ajhg.2017.04.001. PubMed PMID: 28475858; PubMed Central PMCID: PMCPMC5420354.

3. Sosnay PR, Siklosi KR, Van Goor F, Kaniecki K, Yu H, Sharma N, et al. Defining the disease liability of variants in the cystic fibrosis transmembrane conductance regulator gene. Nature Genetics. 2013;45(10):1160-1167. doi: 10.1038/ng.2745.

4. Gottschalk LB, Vecchio-Pagan B, Sharma N, Han ST, Franca A, Wohler ES, et al. Creation and characterization of an airway epithelial cell line for stable expression of CFTR variants. J Cyst Fibros. 2016;15(3):285-294. doi: 10.1016/j.jcf.2015.11.010. PubMed PMID: 26694805; PubMed Central PMCID: PMCPMC4879073.

5. Krasnov KV, Tzetis M, Cheng J, Guggino WB, Cutting GR. Localization studies of rare missense mutations in cystic fibrosis transmembrane conductance regulator (CFTR) facilitate interpretation of genotype-phenotype relationships. Hum Mutat. 2008;29(11):1364-1372.
