# Supplementary figures and images for: Capitalizing on the heterogeneous effects of CFTR nonsense and frameshift variants to inform therapeutic strategy for cystic fibrosis
Source: PLoS Genet. 2018 Nov 16;14(11):e1007723. doi: 10.1371/journal.pgen.1007723 (PMC6267994; doi:10.1371/journal.pgen.1007723)

## Slide 1
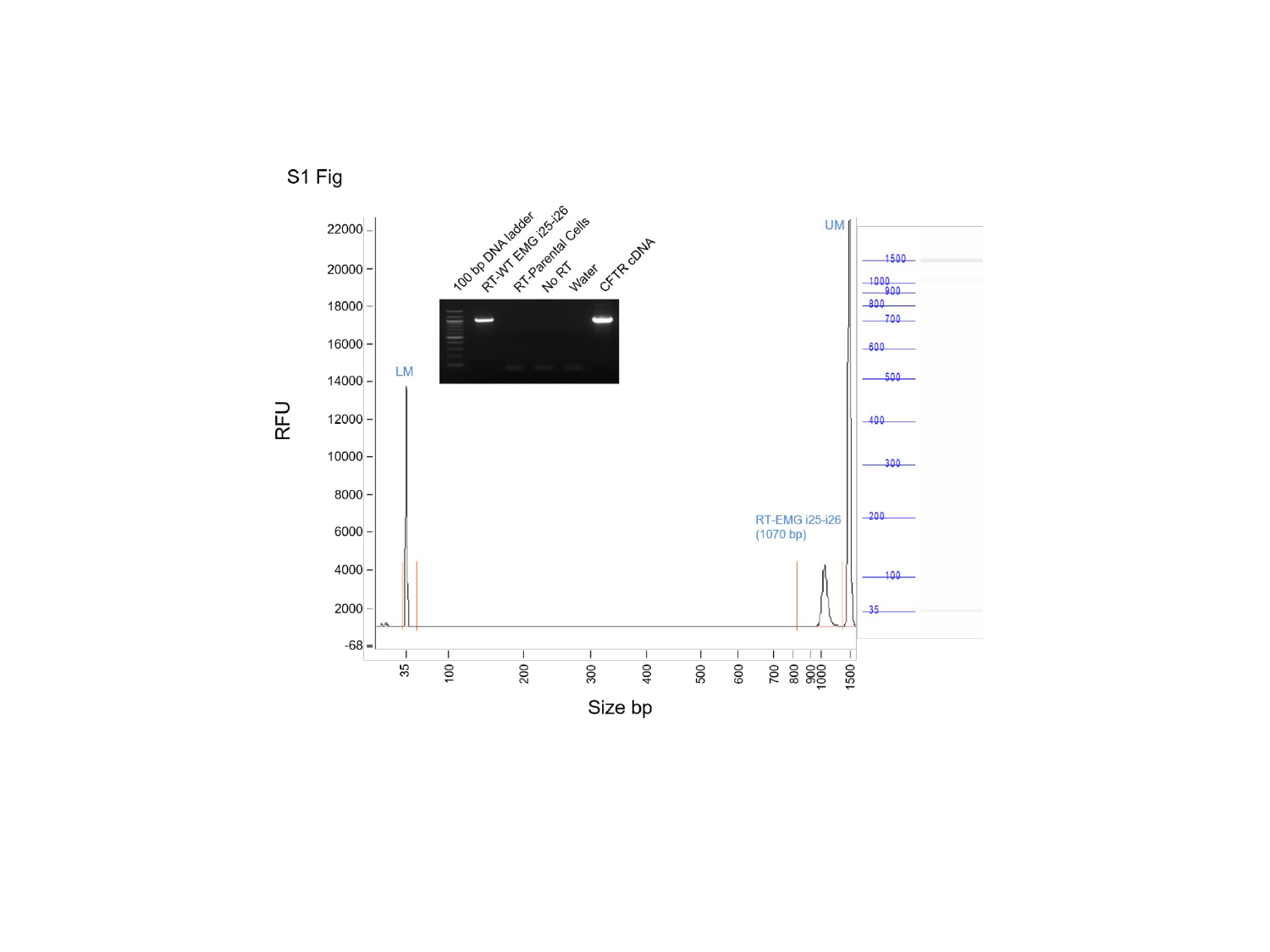

Supplement: S1 Fig — Inset shows agarose gel electrophoresis. Plasmid harboring intronless full-length CFTR was used a positive control. Samples with no RT, water control, and parental cells that lack endogenous CFTR expression were used as negative controls. Automated sizing of DNA fragment was performed by the electrophoresis of RT-PCR product on Fragment Analyzer Automated CE System using 35 bp-1500 bp size standards available from Advanced Analytical Technologies. UM indicates upper marker and LM indicates lower marker. RFU refers to Relative Fluorescence Units. (PPTX) [file pgen.1007723.s002.pptx]

S2 Fig

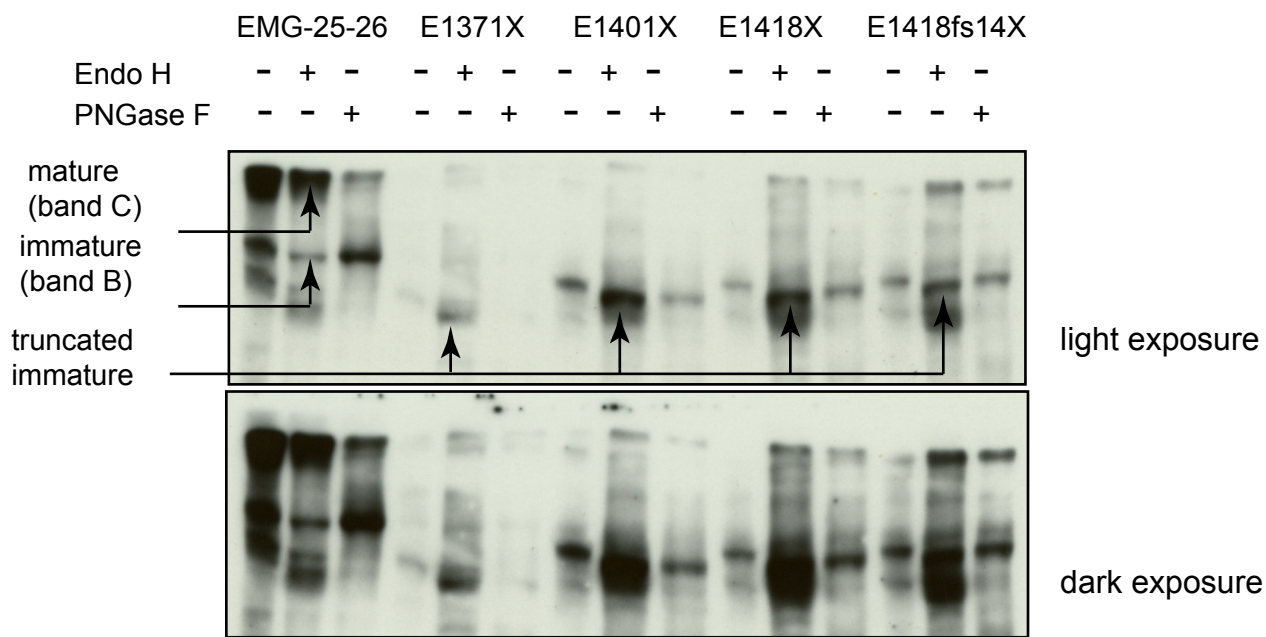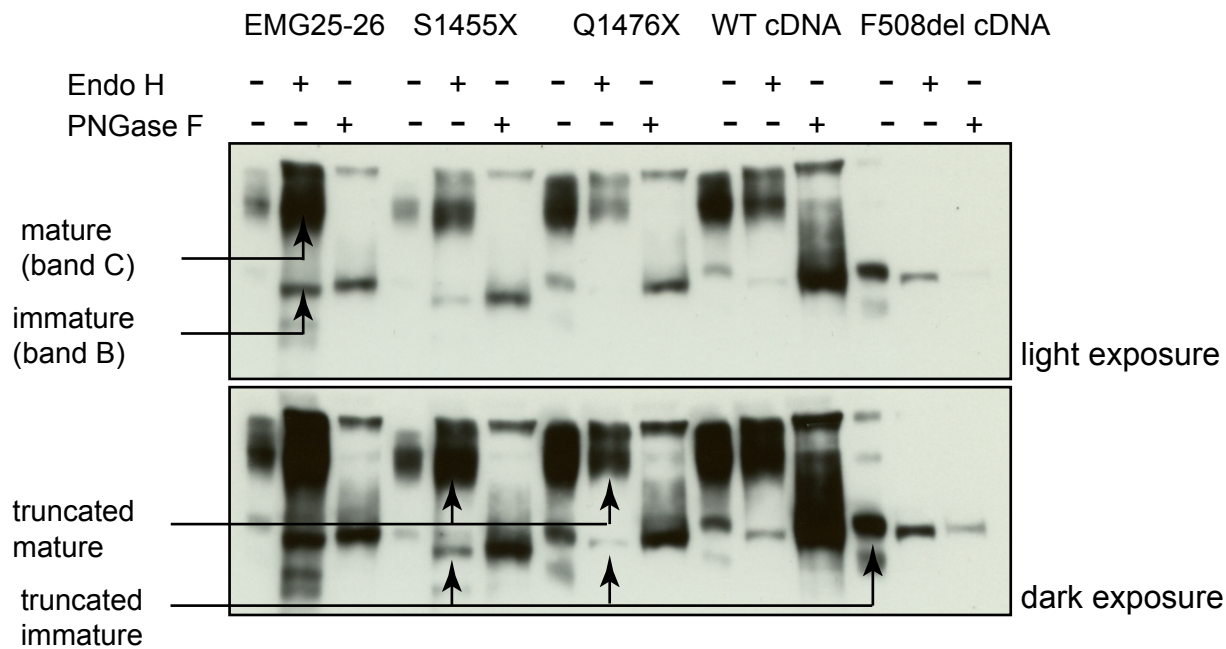

Supplement: S2 Fig — Mature complex glycosylated band is sensitive to PNGase F only, whereas immature core glycosylated band is sensitive to both PNGase F and Endo H. Whole lysates were collected from HEK293 cells expressing WT-EMG or EMGs with different PTC-generating variants. Deglycosyation was achieved by Endo H and PNGase F following manufacturer’s protocol (New England Biolabs), except that denaturation was performed at 37°C. Fifty microgram of total cell lysate was used for deglycosylation followed by electrophoresis. Respective undigested lysates (30 μg) were used as controls. Lysates from cells expressing either intronless WT-CFTR or F508del served as additional controls. IB was probed with anti-CFTR antibody (596 # Cystic Fibrosis Foundation Therapeutics). Arrows indicate mature and immature forms of either full-length or truncated CFTR. Both light and dark exposures are provided. (PDF) [file pgen.1007723.s003.pdf]

## Slide 1
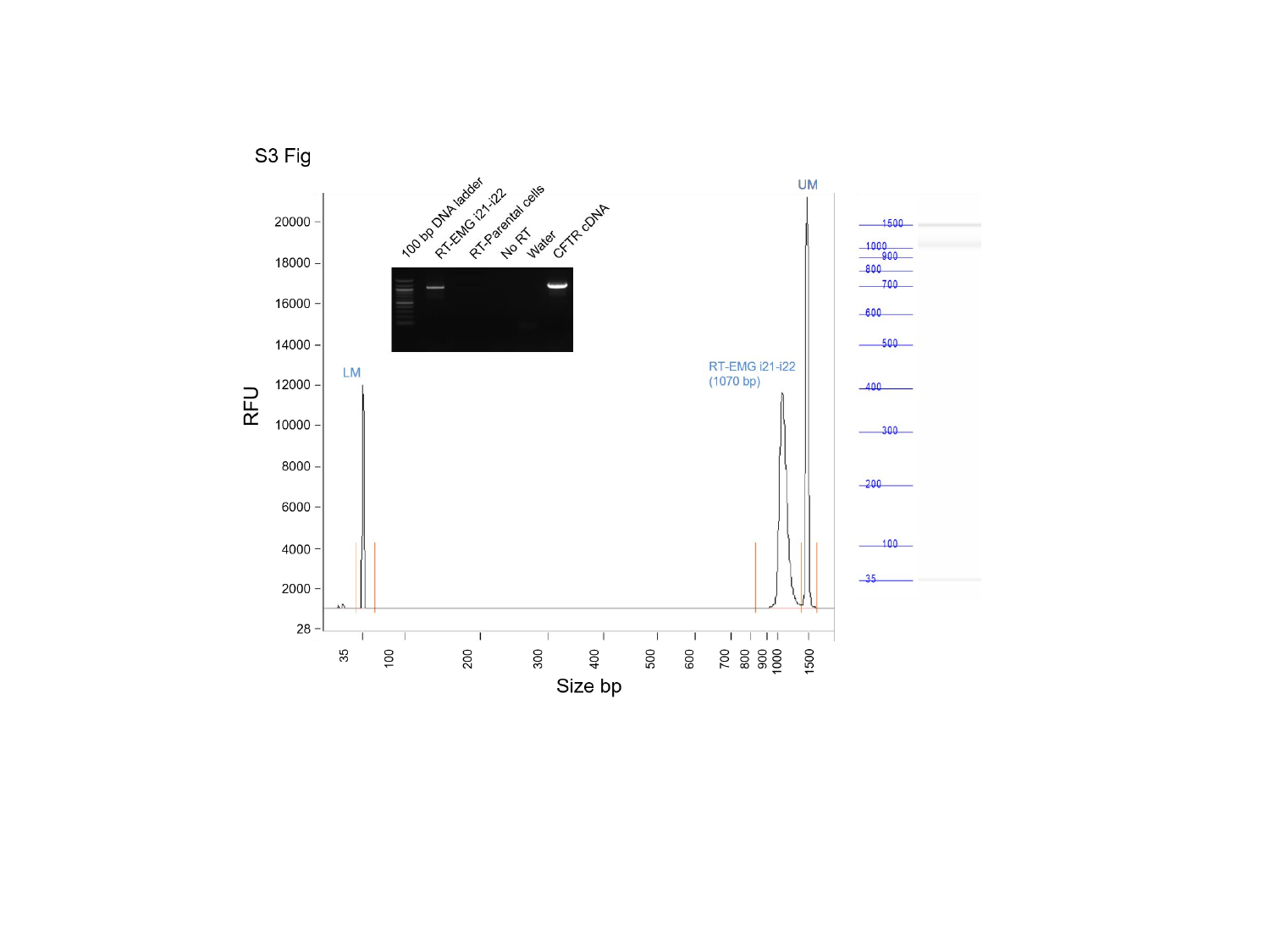

Supplement: S3 Fig — Inset shows agarose gel electrophoresis. A single nucleotide alteration c.3519T>G (p.Gly1173Gly) was introduced to avoid missplicing of EMG-i21-22. Plasmid harboring intronless full-length CFTR was used a positive control. Samples with no RT, water control, and parental cells that lack endogenous CFTR expression were used as negative controls. Automated sizing of DNA fragment was performed by the electrophoresis of RT-PCR product on Fragment Analyzer Automated CE System using 35 bp-1500 bp size standards available from Advanced Analytical Technologies. UM indicates upper marker and LM indicates lower marker. RFU refers to Relative Fluorescence Units. (PPTX) [file pgen.1007723.s004.pptx]

## Slide 1
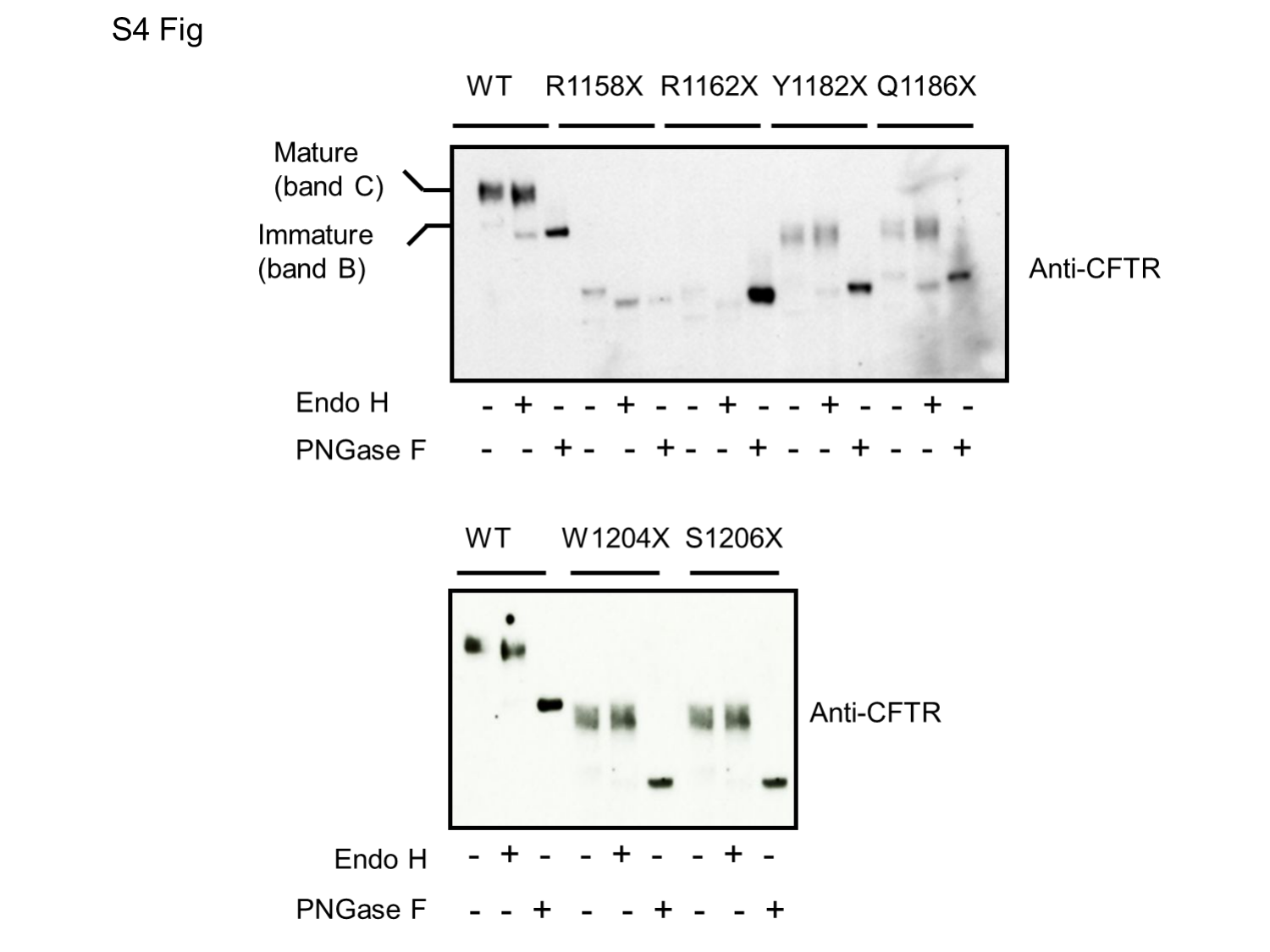

Supplement: S4 Fig — Mature complex glycosylated band is sensitive to PNGase F only, whereas immature core glycosylated band is sensitive to both PNGase F and Endo H. IB was probed with anti-CFTR antibody-MM13-4 (EMD Millipore). (PPTX) [file pgen.1007723.s005.pptx]

## Slide 1
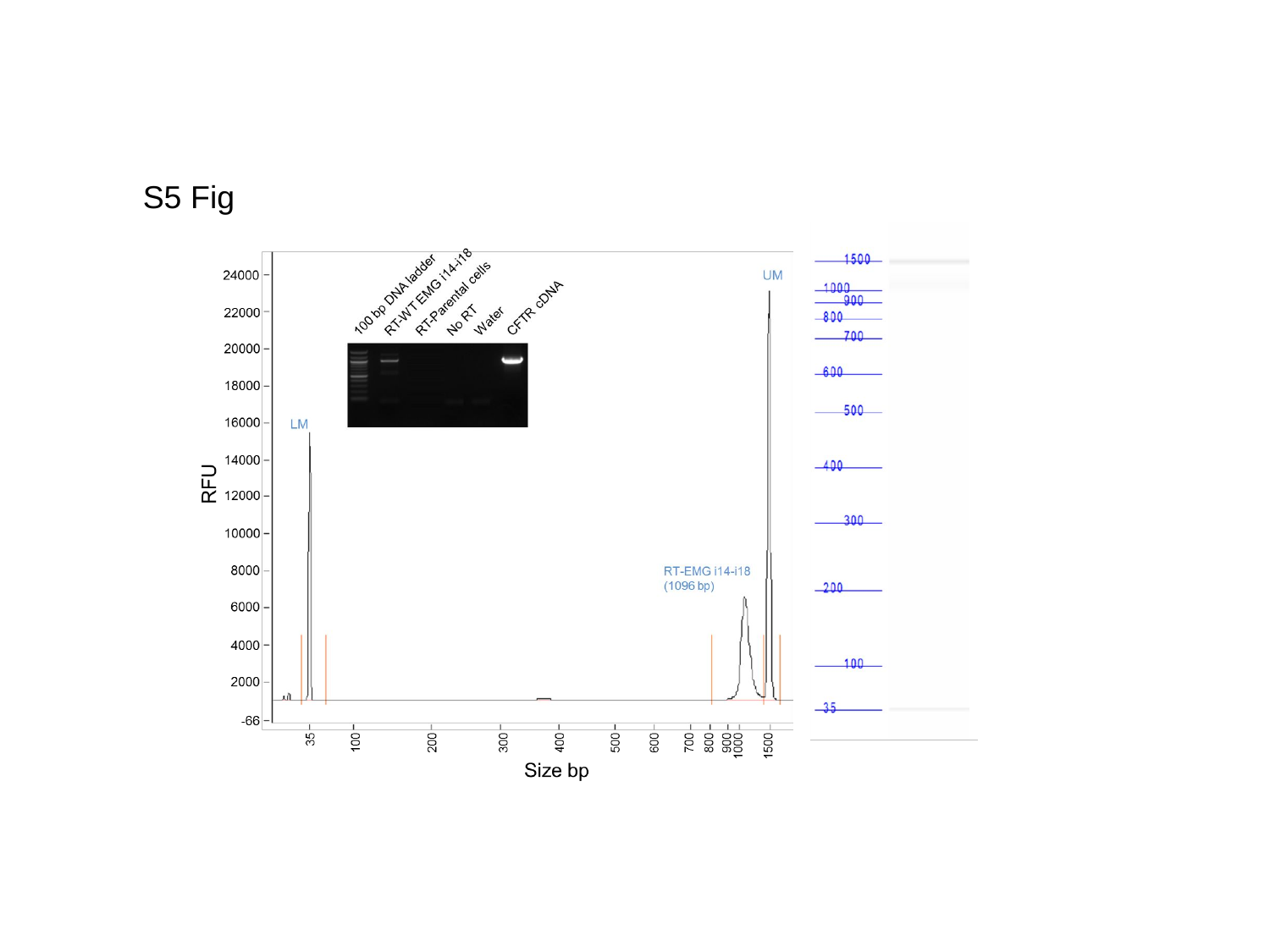

S5 Fig

Supplement: S5 Fig — Inset shows agarose gel electrophoresis. Plasmid harboring intronless full-length CFTR was used a positive control. Samples with no RT, water control, and parental cells that lack endogenous CFTR expression were used as negative controls. Automated sizing of DNA fragment was performed by the electrophoresis of RT-PCR product on Fragment Analyzer Automated CE System using 35 bp-1500 bp size standards available from Advanced Analytical Technologies. UM indicates upper marker and LM indicates lower marker. RFU refers to Relative Fluorescence Units. (PPTX) [file pgen.1007723.s006.pptx]

## Slide 1
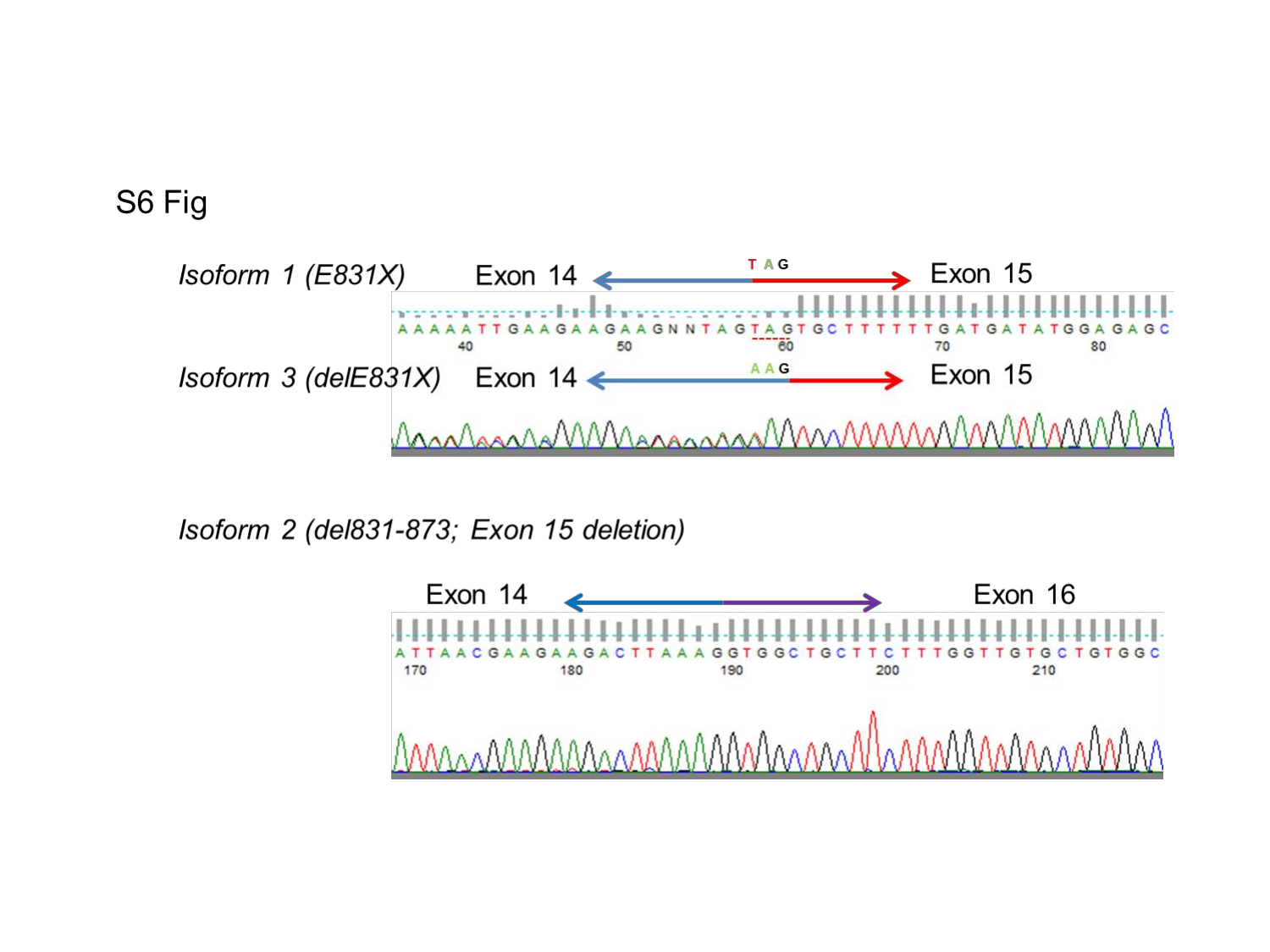

Supplement: S6 Fig — Total RNA was isolated from HEK293 cells stably expressing EMG-i14-18-E831X. RT-PCR was performed using CFTR specific primers. (PPTX) [file pgen.1007723.s007.pptx]

## Slide 1
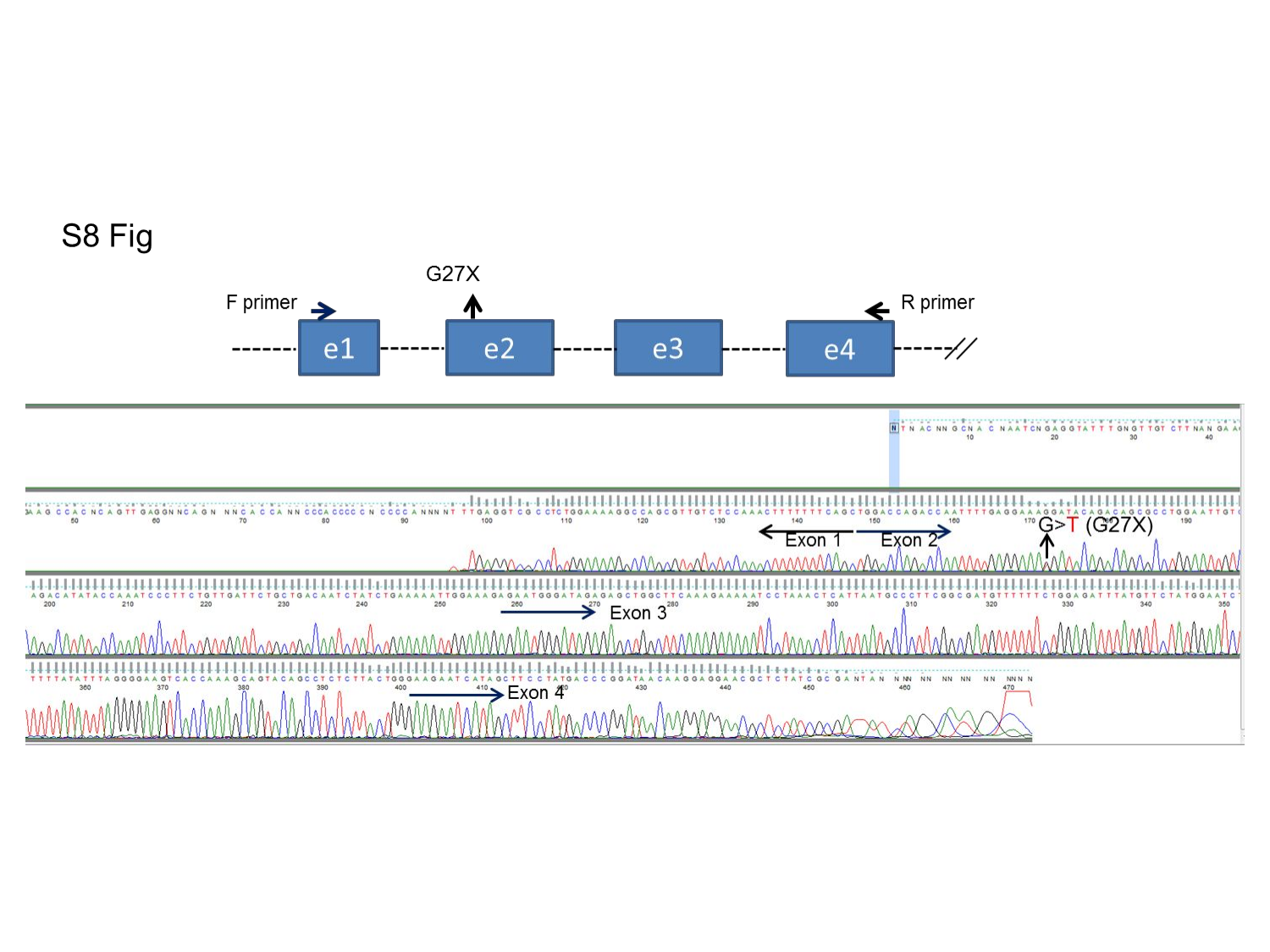

Supplement: S8 Fig — Illustration on the top shows location of CFTR-G27X variant in the exon 2 indicated by vertical arrow. Horizontal arrows indicate location of CFTR specific forward and reverse primers used in the RT-PCR. (PPTX) [file pgen.1007723.s009.pptx]

## Slide 1
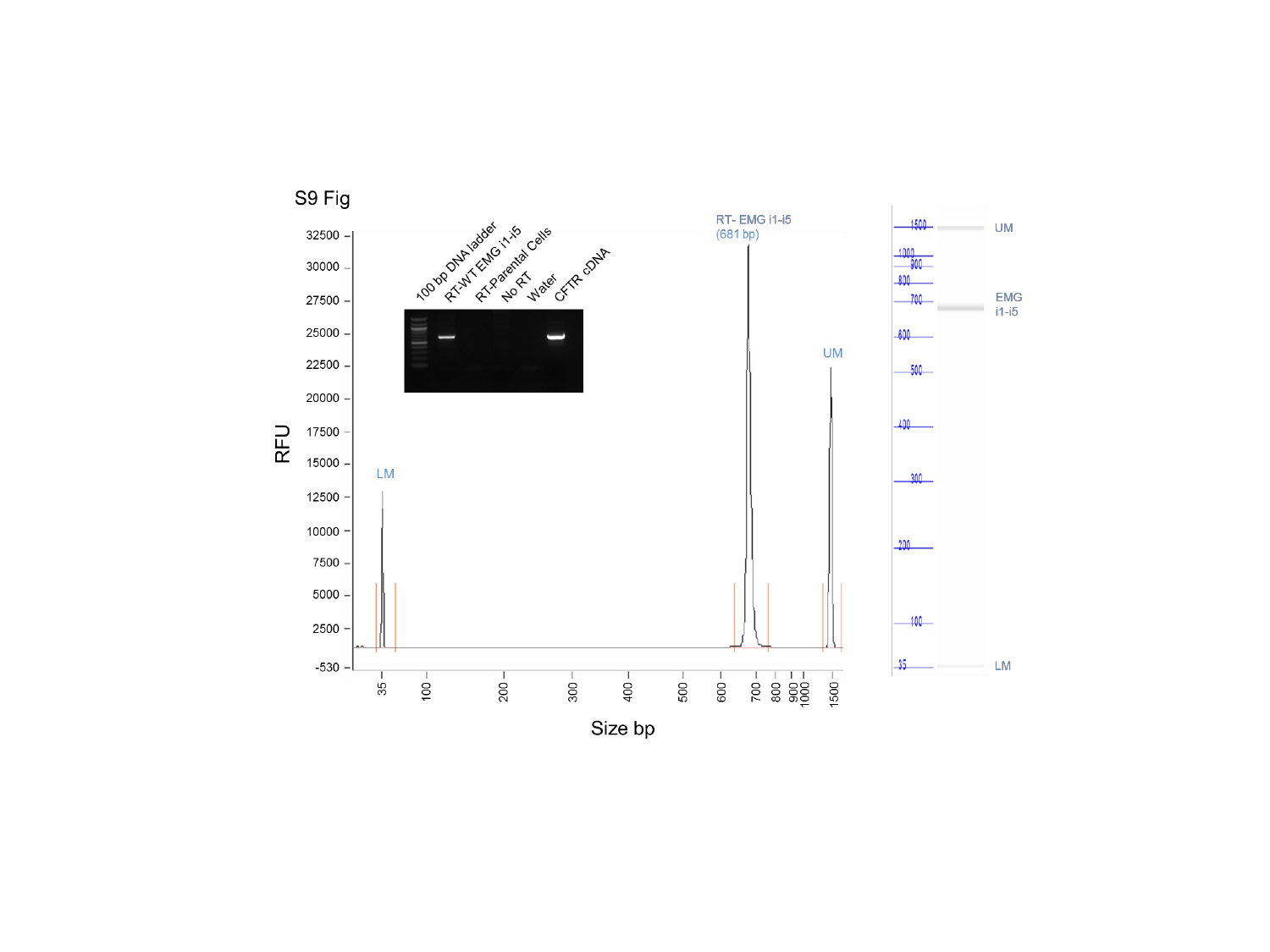

Supplement: S9 Fig — Inset shows agarose gel electrophoresis. Plasmid harboring intronless full-length CFTR was used a positive control. Samples with no RT, water control, and parental cells that lack endogenous CFTR expression were used as negative controls. Automated sizing of DNA fragment was performed by the electrophoresis of RT-PCR product on Fragment Analyzer Automated CE System using 35 bp-1500 bp size standards available from Advanced Analytical Technologies. UM indicates upper marker and LM indicates lower marker. RFU refers to Relative Fluorescence Units. (PPTX) [file pgen.1007723.s010.pptx]

## Slide 1
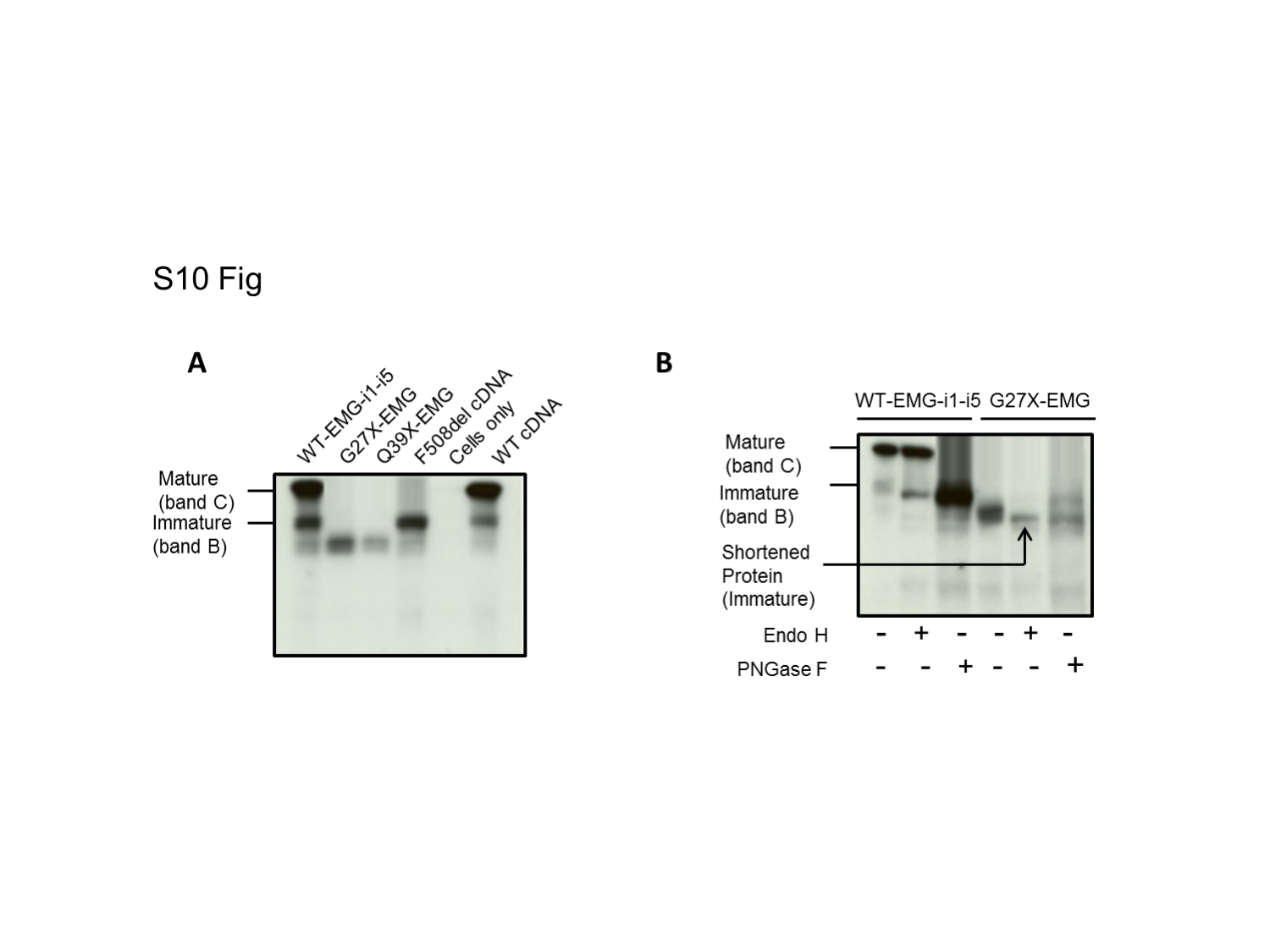

Supplement: S10 Fig — (A) Immunoblot of the naturally occurring 5’-truncations on the steady state amounts of CFTR protein expressed in HEK293 cells. CFTR was visualized with anti-CFTR antibody-596 (CFFT). (B) Representative IB showing sensitivity of CFTR to PNGase F and Endo H. Mature complex glycosylated band is sensitive to PNGase F only, whereas immature core glycosylated band is sensitive to both PNGase F and Endo H. Fifty microgram of total cell lysate was used for deglycosylation followed by electrophoresis. Respective undigested lysates (30 μg) were used as controls. IB was probed with anti-CFTR antibody (596 # Cystic Fibrosis Foundation Therapeutics). Arrow indicates immature form of shortened CFTR produced from EMG i1-i5 harboring G27X. (PPTX) [file pgen.1007723.s011.pptx]
