# Supplementary material for: Capitalizing on the heterogeneous effects of CFTR nonsense and frameshift variants to inform therapeutic strategy for cystic fibrosis
Source: PLoS Genet. 2018 Nov 16;14(11):e1007723. doi: 10.1371/journal.pgen.1007723 (PMC6267994; doi:10.1371/journal.pgen.1007723)
Supplement: S7 Fig — (A) Heat map showing relative expression of CFTR and genes implicated in NMD. Housekeeping genes (B2M, GAPDH, and TBP) are shown as controls. (B) Sashimi plots showing exon 3 harboring L88X variant is normal spliced. Per-base expression is plotted on y-axis of Sashimi plot, genomic coordinates on x-axis, and spliced mRNA are shown on bottom (exons in black, introns as lines with arrow heads). RNA from healthy individual was used as control. (C) Splicing patterns in B2M from both L88X/F508del and healthy individual are shown as controls. (PPTX) [file pgen.1007723.s008.pptx]

## Slide 1
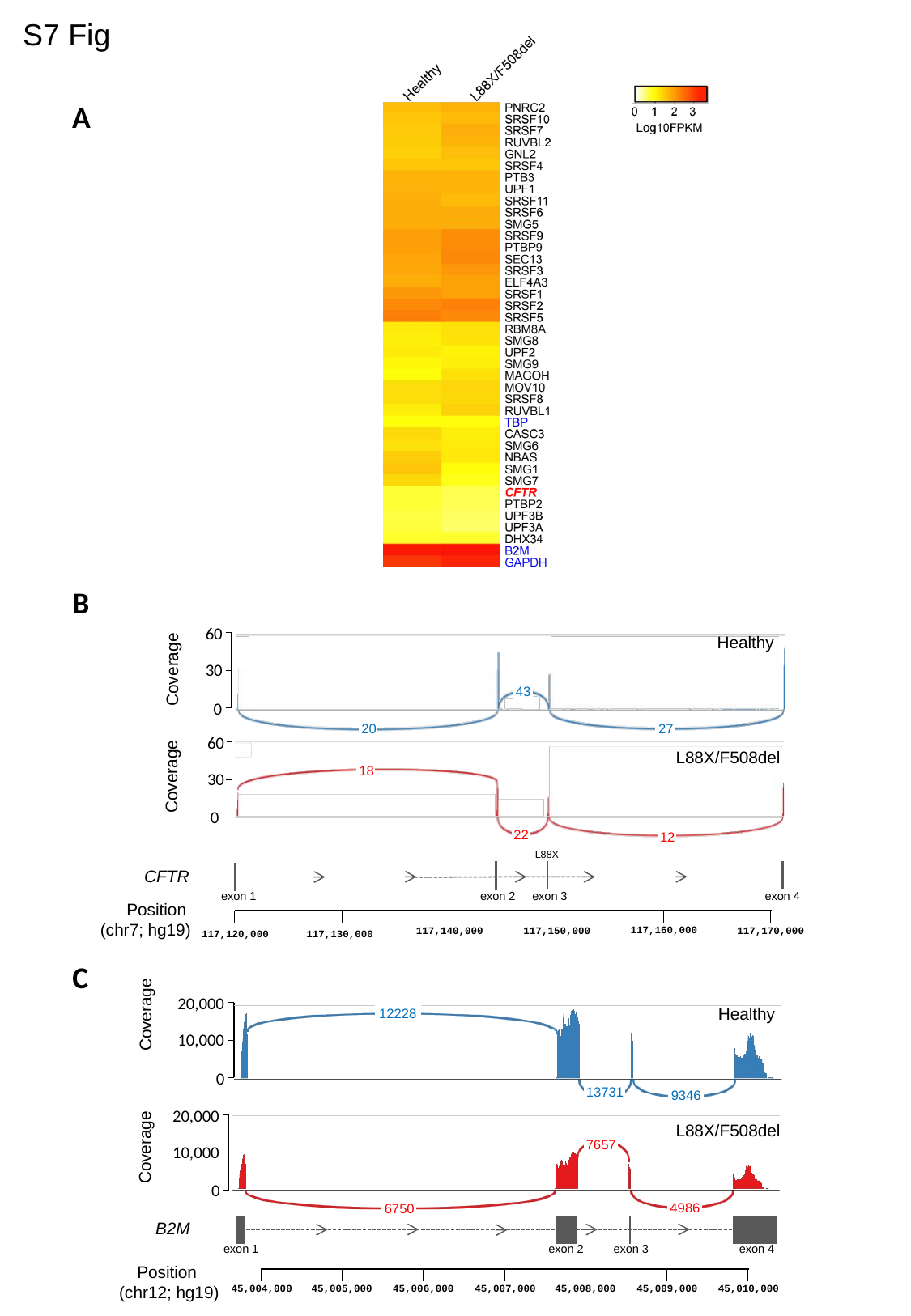

S7 Fig
A
B
60
30
Healthy
Coverage
43
0
20
27
60
30
L88X/F508del
18
Coverage
0
22
12
L88X
CFTR
exon 1
exon 2
exon 3
exon 4
Position
(chr7; hg19)
117,160,000
117,140,000
117,150,000
117,170,000
117,120,000
117,130,000
C
20,000
10,000
Healthy
Coverage
12228
0
13731
9346
20,000
10,000
L88X/F508del
7657
Coverage
0
4986
6750
B2M
exon 1
exon 2
exon 3
exon 4
Position
(chr12; hg19)
45,004,000
45,005,000
45,006,000
45,007,000
45,008,000
45,009,000
45,010,000
